# *Ascl1* as a Novel Player in the *Ptf1a* Transcriptional Network for GABAergic Cell Specification in the Retina

**DOI:** 10.1371/journal.pone.0092113

**Published:** 2014-03-18

**Authors:** Nicolas Mazurier, Karine Parain, Damien Parlier, Silvia Pretto, Johanna Hamdache, Philippe Vernier, Morgane Locker, Eric Bellefroid, Muriel Perron

**Affiliations:** 1 UPR Centre national de la recherche scientifique 3294 Neurobiology and Development, University Paris Sud, Orsay, France; 2 ULB-IBMM, Laboratoire d'Embryologie Moléculaire, Gosselies, Belgium; 3 UPR Centre national de la recherche scientifique 3294 Neurobiology and Development, Institut A. Fessard, Gif-Sur-Yvette, France; Indiana University, United States of America

## Abstract

In contrast with the wealth of data involving bHLH and homeodomain transcription factors in retinal cell type determination, the molecular bases underlying neurotransmitter subtype specification is far less understood. Using both gain and loss of function analyses in *Xenopus*, we investigated the putative implication of the bHLH factor Ascl1 in this process. We found that in addition to its previously characterized proneural function, Ascl1 also contributes to the specification of the GABAergic phenotype. We showed that it is necessary for retinal GABAergic cell genesis and sufficient in overexpression experiments to bias a subset of retinal precursor cells towards a GABAergic fate. We also analysed the relationships between Ascl1 and a set of other bHLH factors using an *in vivo* ectopic neurogenic assay. We demonstrated that *Ascl1* has unique features as a GABAergic inducer and is epistatic over factors endowed with glutamatergic potentialities such as *Neurog2*, *NeuroD1* or *Atoh7*. This functional specificity is conferred by the basic DNA binding domain of Ascl1 and involves a specific genetic network, distinct from that underlying its previously demonstrated effects on catecholaminergic differentiation. Our data show that GABAergic inducing activity of Ascl1 requires the direct transcriptional regulation of *Ptf1a*, providing therefore a new piece of the network governing neurotransmitter subtype specification during retinogenesis.

## Introduction

During development, neural specification leads to the emergence of a large diversity of neuronal subtypes that will serve distinct functions in the adult nervous system. A key issue concerns the nature and action of the molecular cues underlying the acquisition of both generic and specific characteristics of neurons. A small number of basic helix-loop-helix (bHLH) transcription factors are necessary and sufficient for progenitor cell commitment towards a neuronal lineage at the expense of a glial fate and have consequently been qualified as “proneural genes” [1]. Beside their generic function, several studies have shown that proneural genes also display context-dependent effects contributing to the differentiation of particular neuronal subtypes [1], [2]. This is the case for the *atonal-related neurogenin* genes (*Neurog1* and *Neurog2*) and the *achaete scute* gene *Ascl1* (*achaete-scute complex homolog 1*; also called *Mash1* in mouse or *Xash1* in *Xenopus*). These genes are mostly expressed in complementary patterns in the murine central and peripheral nervous system where they drive the production of distinct neuronal populations [3], [4], [5], [6]; reviewed in [1], [7], [8]. In particular, *Neurog2* and *Ascl1* appear respectively as key regulators of glutamatergic (excitatory) *versus* GABAergic (inhibitory) neuronal fates during telencephalic development [4], [6], [9], [10], [11], [12]. The implication and requirement of *Ascl1* in GABAergic cell specification has however proved to be highly time and space dependent [10], [13], [14]. In addition, *Ascl1* is also involved in the development of other neuronal subtypes such as hindbrain serotonergic neurons [15], [16], central and peripheral noradrenergic neurons [17], [18], [19] or mesencephalic dopaminergic neurons [20]. Exploring its involvement in other nervous system regions, such as the retina, and unravelling how it integrates within distinct genetic networks are now required to better understand its divergent properties.

In the retina, beside its proneural function [21], [22], [23], *Ascl1* may also have a more specialized role in the commitment of particular cell subtypes since its expression is restricted to subsets of neuronal progenitors, mostly distinct here again from the *Neurog2*-expressing ones [3], [24], [25], [26]. If numerous studies have addressed the intrinsic mechanisms responsible for the generation of the diverse classes of retinal neurons such as photoreceptor or ganglion cells [27], [28], [29], studies reporting on the molecular cues governing the specification of retinal subtypes with a specific neurotransmitter phenotype remain sparse [29], [30], [31], [32], [33], [34]. As in the brain, GABAergic and glutamatergic neurons represent the most abundant inhibitory and excitatory neurons of the retina, respectively. They are distributed among the 6 major classes of retinal neurons: photoreceptor, bipolar and ganglion cells are mainly glutamatergic, whereas most GABAergic retinal neurons are found within amacrine and horizontal cells. Only a few factors, such as the bHLH transcription factor BHLHB5 [35], the cofactor LMO4 [36], the orphan nuclear receptor Nr4a2 [37] or the PACAP receptor PAC1 [38] have so far been involved in GABAergic amacrine cell production in the retina.

Our previous studies and others highlighted that the bHLH gene *Ptf1a* (*pancreas transcription factor 1a*), drives inhibitory neuron commitment in the retina, at the expense of a glutamatergic destiny [39], [40], [41], [42], [43]. However, upstream regulators, partners and targets of *Ptf1a* within the retina remain to be investigated. Considering the GABAergic instructive function of *Ascl1* in various regions of the brain, and the fact that *Ptf1a* expression is decreased in the dorsal spinal cord and retina of *Ascl1^−/−^* mice [14], [21], we asked whether it could be required for retinal GABAergic cell specification and whether it could take part to the *Ptf1a* transcriptional network.

To address these issues, we first analysed *Ascl1* function during *Xenopus* retinogenesis. We found that *Ascl1* is required for GABAergic retinal neuron genesis and sufficient to bias a subset of retinal progenitors towards a GABAergic destiny. Then, we took advantage of the *Xenopus* ectopic neurogenesis induction paradigm to investigate which genetic interactions contribute to Ascl1 activity as an inducer of neurotransmitter phenotypes. We showed that distinct *Ascl1*-dependent transcriptional networks sustain the production of GABAergic and catecholaminergic neurons. Together, our data suggest that the GABAergic activity of Ascl1 requires *Ptf1a*, this latter being a direct transcriptional target of Ascl1.

## Materials and Methods

### Constructs

pCS2-Ascl1 (also called Xash1 [44]), pCS2-Ptf1a, pCS2-Ptf1a-GR [45], pCS2-XNeurog2 (also called XNgnr-1), pCS2-Neurog2-GR [46], pCS2-Atoh7 (also called Xath5 [47]), pCS2-NeuroD1 (also called NeuroD [48]), pCS2-GFP [49] and all glucocorticoid-inducible chimeric Ascl1:Neurog2 constructs [50] have previously been described. pCS2-flag-Ascl1-GR was generated by subcloning the Ascl1 coding sequence from pCS2-NLSMT plasmid into EcoRI and XhoI sites of pCS2-flag-GR [51]. Protein activity of GR constructs was induced by addition of 4 μg/ml dexamethasone (dex, Sigma).

### Embryos, *in vitro* RNA synthesis and microinjection

*Xenopus laevis* embryos (up to stage 41) were obtained by conventional procedures of *in vitro* fertilization and staged according to Nieuwkoop and Faber [52]. At the desired stage, embryos were fixed in 4% paraformaldehyde. Capped sense mRNAs were prepared from CS2 plasmids after Not1 digestion and transcribed using the mMessage mMachine SP6 kit (Ambion). mRNAs were then purified with Sephadex Column (Roche). 100–150 pg of mRNAs were injected into one or two blastomeres at the two- or four-cell stage. *GFP* mRNA was co-injected as a tracer. Loss of function experiments were performed using already described and validated antisense oligonucleotides morpholinos: *Ptf1a*-Mo [39], *Ascl1*-Mo (a mix of two morpholinos, *Ascl1*-Mo1 and *Ascl1*-Mo2, that target the two *Xenopus laevis Ascl1* alloalleles; [53]), *Phox2a*-Mo and *Hand2*-Mo [53]. As a control, standard morpholinos purchased from GeneTools (LLC) were used. 8 ng of morpholinos were injected into one or two blastomeres at the two-cell stage. All embryos were co-injected with *GFP* mRNA as a tracer and only embryos exhibiting GFP fluorescence in the eye were selected for further analysis.

### *In vivo* lipofection

*pCS2-Ascl1, pCS2-Neurog2, pCS2-Atoh7*, *pCS2-NeuroD1* were transfected in stage 18 neurula into the presumptive region of the retina as previously described [54], [55]. *pCS2-GFP* was co-lipofected and used as a tracer to follow transfected cells. Of note, co-lipofection efficiency was previously shown to be extremely high (85%–100%) [54]. Embryos were fixed at stage 41 in 4% paraformaldehyde plus 0,3% glutaraldehyde and cryostat sectioned (12 μm). Transfected cells were counted and cell types were identified based upon their laminar position and morphology.

### Immunohistochemistry

Immunohistochemistry was performed using rabbit polyclonal anti-GABA (1/1000; ImmunoStar), mouse monoclonal anti-GFP (1/200; Molecular Probes) and anti-mouse or anti-rabbit fluorescent secondary antibodies (1/1000; Alexa, Molecular Probes). Cell nuclei were counterstained with Hoechst (Sigma).

### *In situ* hybridization

Digoxigenin-labeled antisens RNA probes for *gad1, VGlut1* [56], *TH* [53], *tubb2b* [48], *Bru* [57], *dpysl3* [58], *Ptf1a* [45] and *Ascl1* [50] were generated according to the manufacturer’s instruction (Roche). Whole mount *in situ* hybridization [59] and double fluorescent *in situ* hybridizations on cryosections [60] were carried out as previously described.

### RT-qPCR

Stage 19 embryos were treated with cycloheximide (CHX, 10 μg/ml) during 2.5 hours. Total RNA from 8 embryos was then isolated using the Nucleospin RNA XS kit (Macherey Nagel). Reverse transcription was performed using IscriptcDNA Synthesis Kit (Biorad). RNA quality was evaluated using Experion (BioRad). qPCR reactions were performed in triplicate using SsoFast Eva green Supermix (Biorad) on a C1000 Thermal Cycler (CFX96 Real-Time System, Biorad). All values were normalized to the level of the reference gene *ornithine decarboxylase* (*ODC*) using Biorad CFX Manager Software. Primers sequences are: *tubb2b* forward 5′cccgtgccatccttgtggatttt3′, *tubb2b* reverse 5′gcccagttattgccagcaccactt3′, *Ptf1a* forward 5′gccgctcaggaaccccaaca3′, *Ptf1a* reverse 5′ggcagcccgtagtctgggtca3′, *ODC* forward 5′catggcattctccctgaagtacaagaa3′ and ODC reverse 5′ggacagtggtaggggcaagctca3′.

### Western-blot analyses

Total protein lysates were prepared from stage 14 embryos and submitted to western blot analysis using an anti-Myc antibody (Sigma) as previously described [61].

### Image analysis and quantification

Shown in figures are representative data from one experiment that has been performed at least in duplicate. Fluorescent staining was visualized with a M2 Zeiss microscope. Images were captured with a digital camera AxioCam MRc and AxioVision Rel 7.8 software. The quantification of *in situ* hybridization signal intensity in the eyes of whole embryos was quantified using Adobe Photoshop CS4.

### Ethics statement

All animal procedures were conducted under the supervision of several licensed personnel, including the director of research of the CNRS and professor at the university Paris-Sud, with licenses to perform *Xenopus* experimentation (authorization 91-29 and 91-28), in accordance with French government policies. The study was conducted under an institutional license (number B 91-471-102 up to 2012 and C 91-471-102 since 2013). The study protocol was approved by the institutional animal care committee, the Direction Départementale de la Protection des Populations (license B/C 91-471-102).

## Results

### *Ascl1* knockdown impairs retinal GABAergic neuron genesis

**Figure 1:**
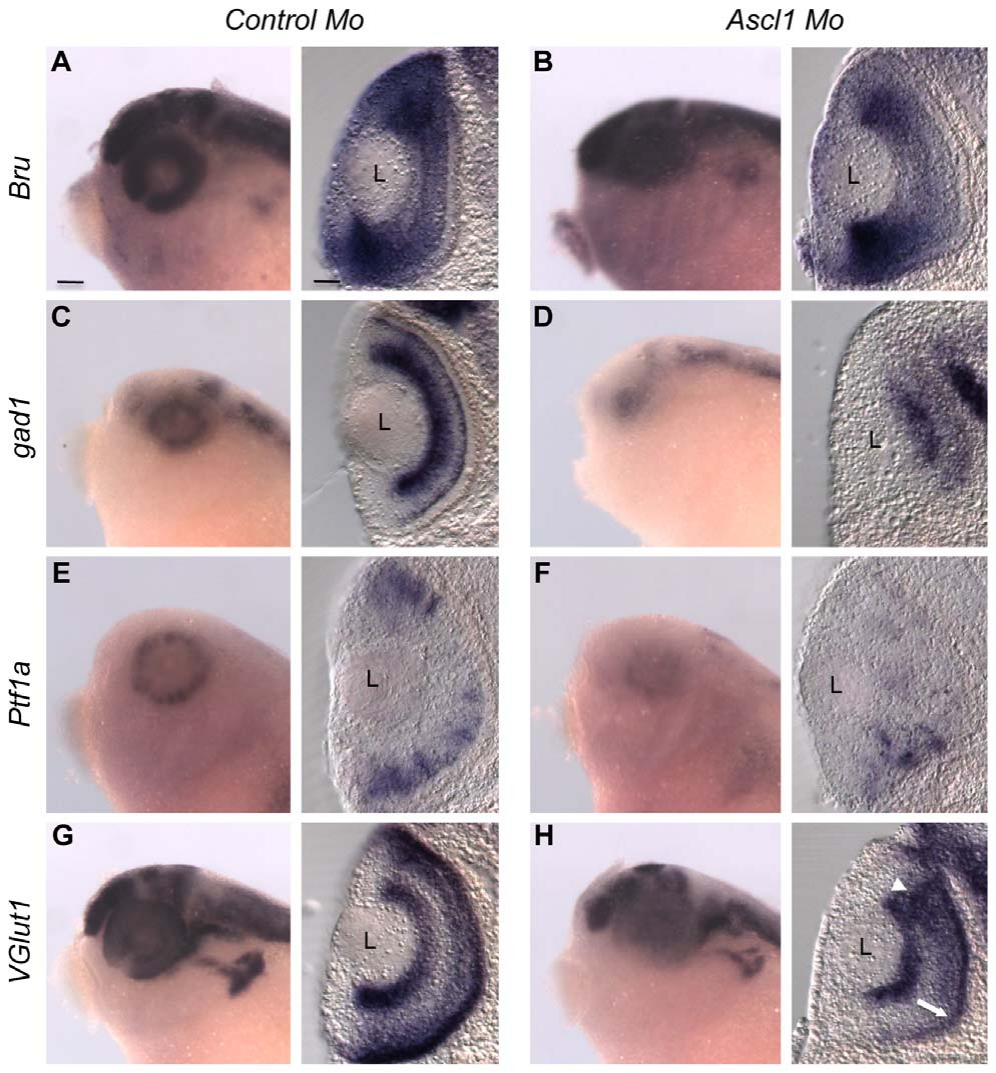
*Ascl1* is required for retinal GABAergic cell genesis. Whole mount *in situ* hybridization on stage 35 embryos following *Ascl1* or control morpholino (Mo) injection in one blastomere at the two-cell stage. Shown for each indicated probe are representative pictures of the observed labelling in the head region (lateral views, anterior on the left) and on retinal cross sections (dorsal side up). The expression of the pan-neuronal marker *Bru* (A, B) is not affected by *Ascl1* knockdown while *gad1* and *Ptf1a* stainings are severely reduced (C-F). *VGlut1* expression (G, H) is unequally affected in the different cell layers: decreased in the photoreceptor layer (white arrow) and extended in the inner nuclear layer (white arrowhead). L: Lens. Scale bar represents 300 μm (heads) or 50 μm (sections).

To address the potential requirement of *Ascl1* in GABAergic and glutamatergic phenotype acquisition, we performed morpholino (Mo) injections in two cell stage embryos. We examined the effect of *Ascl1* loss-of-function by analysing the expression of *gad1* (*glutamic acid decarboxylase*) and *VGlut1* (*vesicular glutamate transporter 1*), which respectively encode the rate-limiting enzyme for GABA biosynthesis and a glutamate transporter expressed in neurons. The expression of a retinal pan-neuronal marker *Bru* [57] remained largely unaffected in *Ascl1*-Mo injected retinas compared to control ones (80% unaffected, n = 10 embryos; Fig. 1A, B), suggesting that *Ascl1* knockdown does not significantly impair neuronal commitment within the retina, as shown in the mouse [23]. *Ascl1* inhibition however caused a marked reduction of *gad1* retinal expression (100% reduced, n = 10 embryos; Fig. 1C, D), indicative of an impaired production of GABAergic neurons. Importantly, this was accompanied by a significant decrease in the expression of *Ptf1a* (90% reduced, n = 10 embryos; Fig. 1E, F), a crucial determining factor of GABAergic fate in the retina [39], [40], [43]. Then, we observed in *Ascl1*-Mo injected retinas that the different glutamatergic neuronal populations were unevenly affected, with *VGlut1* expression being largely unchanged in ganglion cells, reduced in the photoreceptor layer, while appearing expanded in the inner nuclear layer (INL) (Fig. 1G, H). Thus, in contrast to the *Ptf1a* knockdown phenotype [39], *Ascl1* morphant retinas do not exhibit an overall increase of *VGlut1* staining. As a whole, blocking Ascl1 mainly precludes commitment of precursor cells towards a GABAergic fate. However, the overall observed defects, in particular the loss of some glutamatergic cells in the outer nuclear layer, cannot be simply explained by a switch from GABAergic to glutamatergic cell types. A plausible explanation would be that part of the *Ascl1* knockdown phenotype primarily results from a loss of its proneural activity.

### *Ascl1* overexpression in the retina biases a subset of precursor cells towards a GABAergic destiny

**Figure 2:**
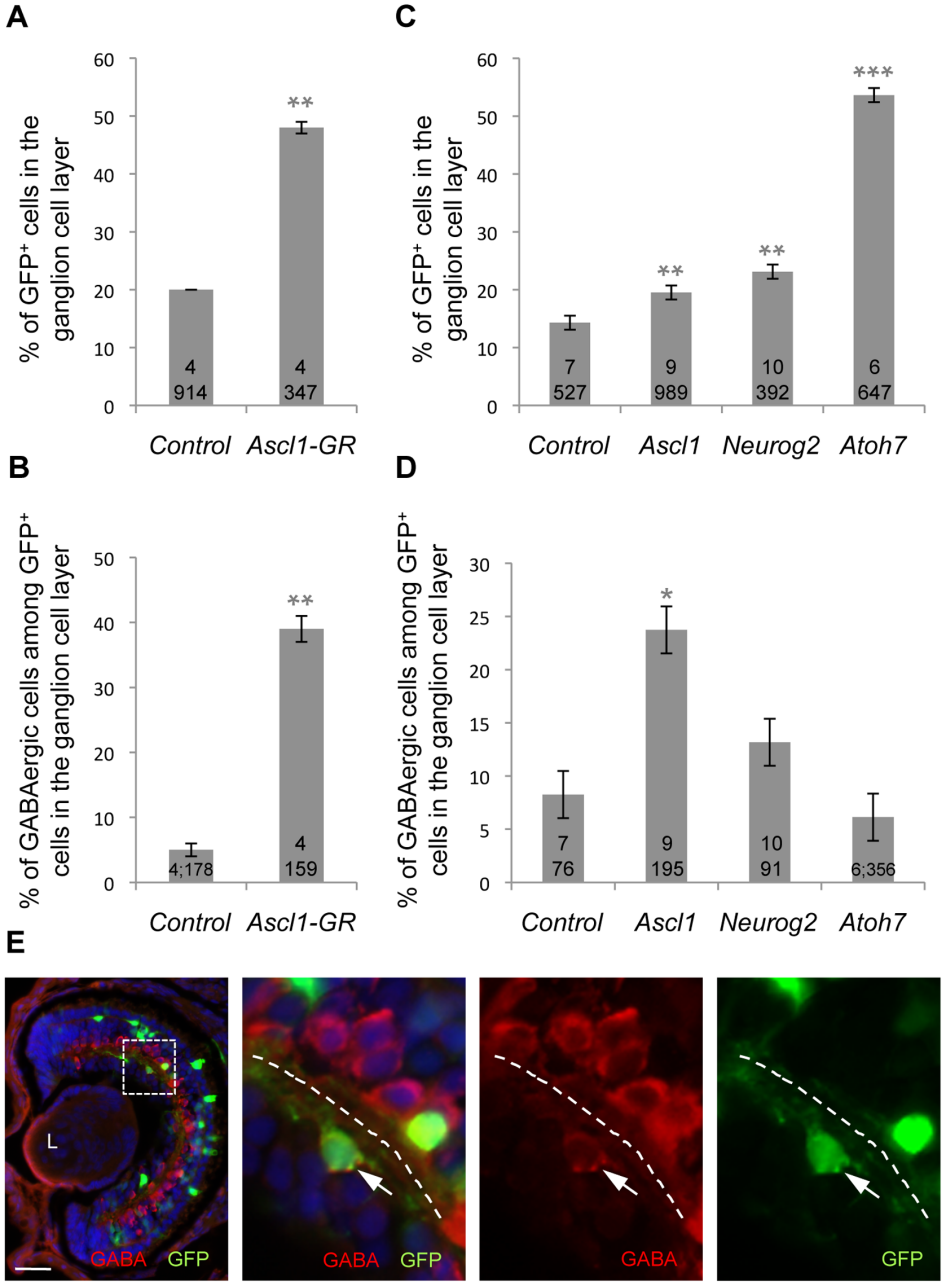
*Ascl1* overexpression in the retina favours GABAergic cell genesis. Cell fate analysis following overexpression of the indicated construct by either mRNA injection in one blastomere at the four-cell stage and dex treatment at stage 16 (A, B; analysis at stage 38) or *in vivo* lipofection at the neurula stage (C, D; analysis at stage 41). In both cases, GFP was used as a tracer to visualize injected/transfected cells. Note that *Ascl1*, *Neurog2* and *Atoh7* lipofections all result in an increased percentage of cells in the ganglion cell layer but only *Ascl1*-overexpressing cells are biased towards a GABAergic destiny. Total number of analyzed retinas and counted cells per condition is indicated in each bar. Values are given as mean +/– s.e.m. p<0,001 (***), p<0,01 (**), p<0,05 (*) (Student’s t-test). (E) shows a typical section of stage 41 retinas lipofected with *GFP* plus *Ascl1* and immunostained with anti-GABA (red) and anti-GFP (green) antibodies. Panels on the right are higher magnifications of the dotted square delineated region. The dotted line indicates the inner plexiform layer, with the ganglion cell layer on the left and the inner nuclear layer on the right. White arrow points to a transfected GABA-positive cell within the ganglion cell layer. L: Lens. Scale bar represents 300 μm (heads) or 50 μm (sections).

To investigate in more detail *Ascl1* involvement in the specification of neurotransmitter phenotypes, we turned to a gain of function strategy using a glucocorticoid-inducible *Ascl1* construct (*Ascl1-GR*), which avoids affecting early nervous system development. We analysed the fate of *Ascl1*-overexpressing cells at stage 38. Contrasting with the *Ptf1a* gain of function, which results in a dramatic overproduction of amacrine and horizontal cells [39], *Ascl1* overexpression led to a typical neurogenic phenotype, with early born neurons being increased (ganglion cells, Fig. 2A) at the expense of late born cells (bipolar and Müller glial cells, data not shown). However, similarly to the *Ptf1a* phenotype, quantification of GABA-positive neurons among *Ascl1*-overexpressing cells revealed an enhanced GABAergic neuronal yield compared to the control situation (Fig. 2B). These supernumerary inhibitory neurons were found within the ganglion cell layer and thus probably correspond to displaced amacrine cells [62]. Importantly, similar results were obtained in a clonal analysis after *in vivo* lipofection (Fig. 2C-E). This rules out the possibility that the *Ascl1* phenotype observed in mRNA injection experiments may be secondary to early morphogenetic defects of the retina and confirms that *Ascl1* exerts a cell-autonomous function in GABAergic cell type determination.

In order to figure out whether this GABAergic inducing activity is specific to Ascl1 compared to other bHLH proteins endowed as well with neurogenic properties, we performed similar *in vivo* lipofection experiments with the *atonal*-related genes *Neurog2* and *Atoh7*. As expected from previous studies [46], [47], [63], overexpression of these genes, as for *Ascl1*, resulted in an imbalanced production of early- *versus* late-born cell types (Fig. 2C and data not shown). However, in contrast to the *Ascl1* gain of function phenotype, the ratio of GABAergic neurons among *Neurog2* or *Atoh7* transfected cells in the ganglion cell layer did not differ from the control situation (Fig. 2D).

Altogether, our data highlight that, although *Ascl1* shares with *Neurog2* or *Atoh7* a neurogenic activity impacting on retinal cell type distribution, it has the specific ability to bias a subset of progenitors towards a GABAergic fate.

### Ascl1 has a specific GABAergic inducing activity and is epistatic to glutamatergic factors

**Figure 3:**
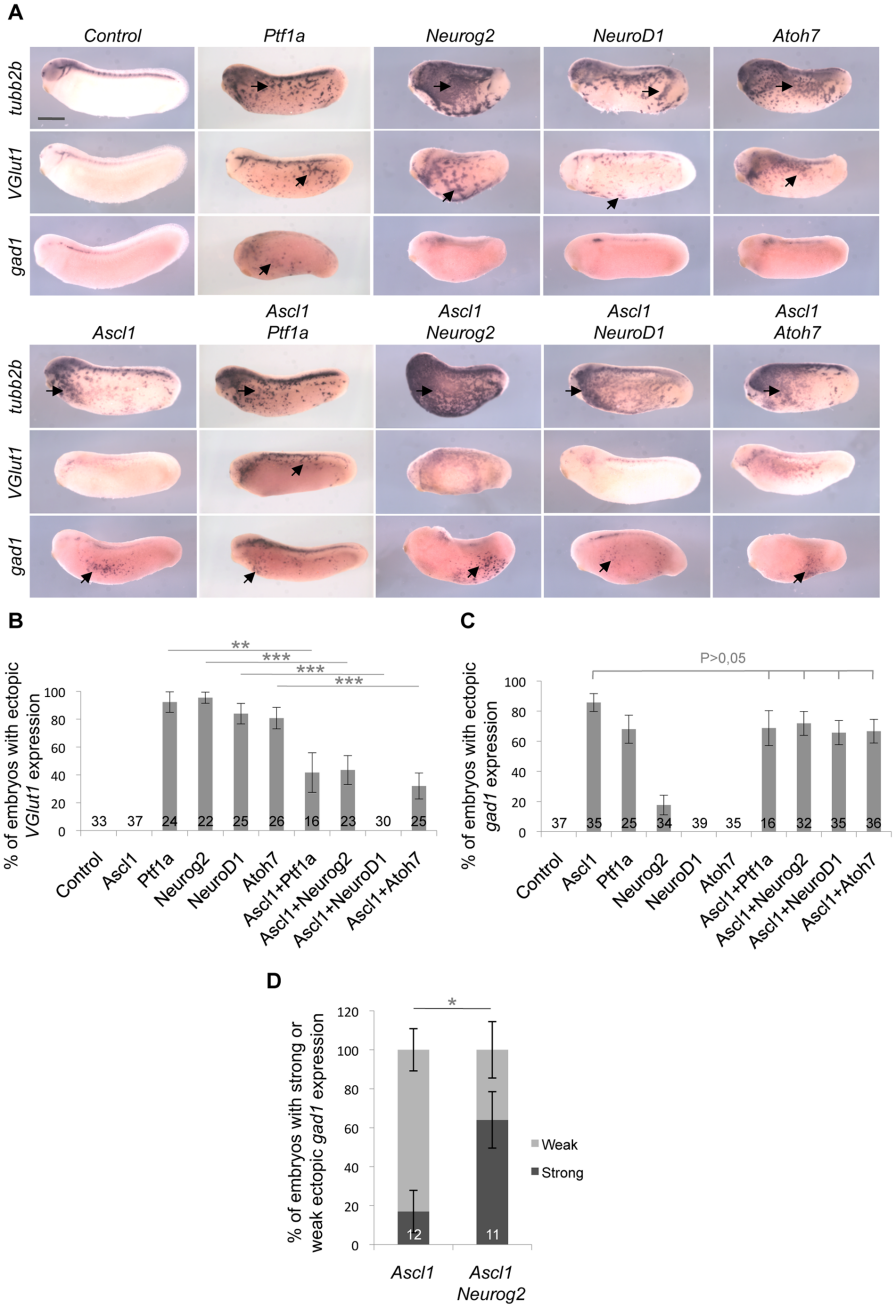
Comparison of GABAergic and glutamatergic inducing activities of five different bHLH factors. Whole mount *in situ* hybridization analyses of *tubb2b, VGlut1* or *gad1* expression on stage 24 embryos injected with the indicated mRNAs in one blastomere at the two-cell stage. (A) Arrows indicate ectopic expression in the epidermis (anterior on the left, dorsal side up). All the tested bHLH factors exhibit neurogenic activity, as inferred from *tubb2b* ectopic expression. However, only *Ascl1* specifically induces *gad1* ^+^ neurons without inducing *VGlut1* ^+^ ones. (B, C) Quantification of embryos displaying *VGlut1* (B) or *gad1* (C) ectopic expression. *Ascl1* interferes with *Ptf1a*-, *Neurog2*-, *NeuroD1*- and *Atoh7*-dependent production of *VGlut1* ^+^ neurons, while none of these factors affect *Ascl1* GABAergic inducing activity. (D) Quantification of embryos with weak or strong ectopic *gad1* staining following mRNA injection of *Ascl1* alone or together with *Neurog2*. Note that *Neurog2* enhances *Ascl1* GABAergic inducing activity. Total number of analyzed embryos per condition is indicated in each bar. Error bars represent 95% confidence intervals. p<0,001 (***), p<0,01 (**), p<0,05 (*) (binomial test). Scale bar represents 500 μm.

It has previously been shown that *Ascl1, Neurog2*, *NeuroD1, Atoh7* and *Ptf1a* are all sufficient to induce ectopic neurogenesis when overexpressed in the epidermis of *Xenopus* embryos [47], [50], [64], [65], [66], [67]. To further assess the functional specificity of *Ascl1* as a GABAergic inducing factor, we injected the corresponding mRNA as well as those encoding the aforementioned bHLH factors and compared the neurotransmitter phenotypes they induced in ectopic neurons formed within the epidermis. As expected, each of them induced ectopic *tubb2b* (previously called *N-tubulin*) expression in stage 24 embryos (Fig. 3A). Except for *Ascl1*, this was associated with a robust *VGlut1* staining (Fig. 3A, B). Only *Ptf1a* and *Ascl1* were able to promote ectopic *gad1* expression (Fig. 3A, C). We therefore conclude that *Ascl1* has the unique property among these factors, to convert presumptive epidermal cells into GABAergic neurons without simultaneously inducing glutamatergic ones.

**Figure 4:**
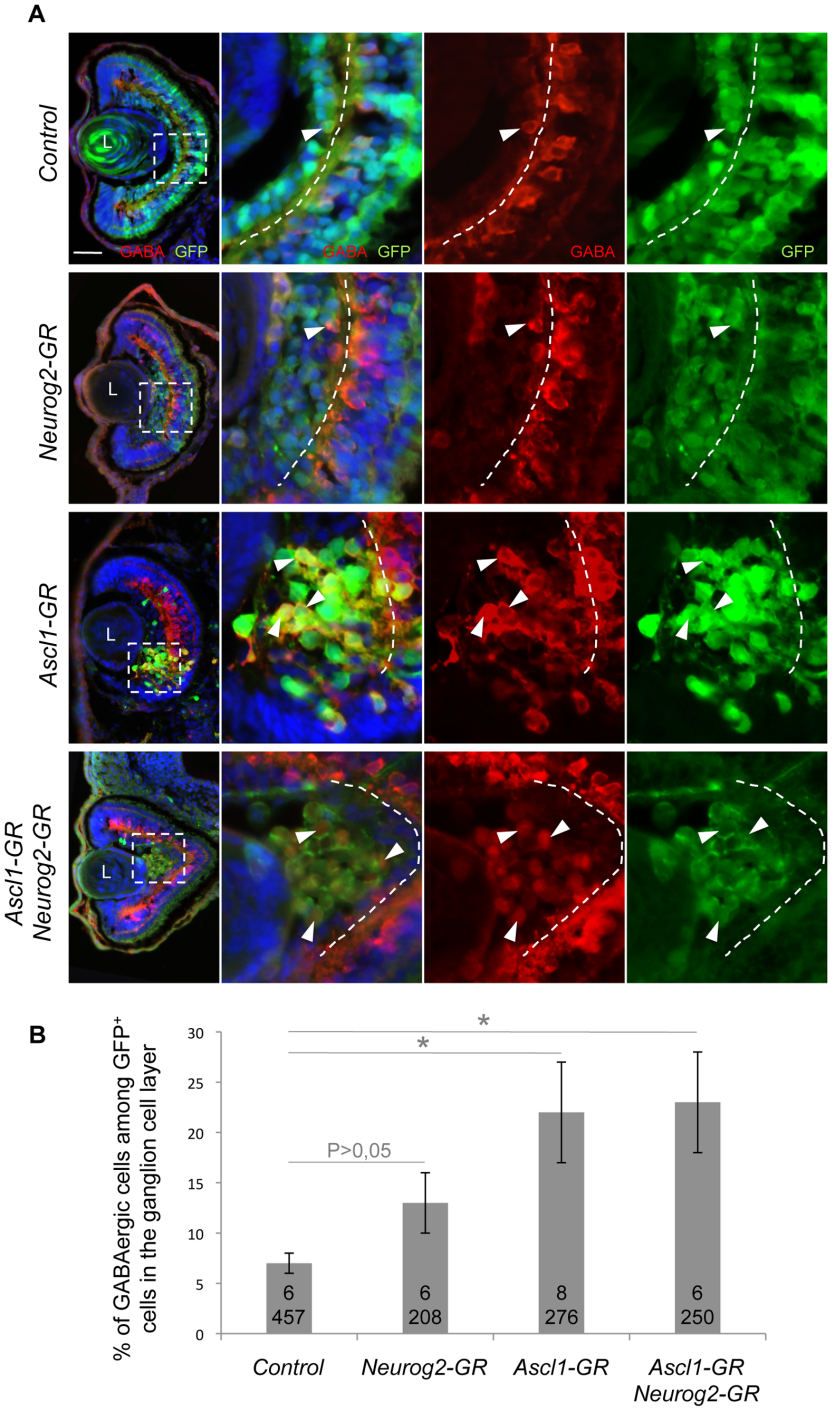
*Ascl1* overexpression together with *Neurog2* still promotes production of GABAergic cells in the retina. (A) Stage 41 retinal sections immunostained with anti-GABA (red) and anti-GFP (green) antibodies. Embryos were injected with the indicated mRNAs in one blastomere at the four-cell stage and treated with dexamethasone at stage 16. Panels on the right are higher magnifications of the dotted square delineated region. The dotted line indicates the inner plexiform layer. Arrowheads point to GFP/GABA-positive cells within the ganglion cell layer. (B) Quantification of GABAergic cell proportion among GFP^+^ cells in the ganglion cell layer, showing that Ascl1 GABAergic inducing activity is not affected by *Neurog2* misexpression. Total number of analyzed retinas and counted cells per condition is indicated in each bar. p<0,05 (*) (Student’s t-test). Values are given +/– s.e.m. L: Lens. Scale bar represents 50 μm.

We next wondered whether Ascl1 might act as an inhibitor of glutamatergic neuron genesis and thus investigated the genetic relationships between *Ascl1* and the other bHLH genes, in co-injection experiments. The glutamatergic inducing activities of *Neurog2*, *NeuroD1, Atoh7* and *Ptf1a* were all dramatically reduced upon *Ascl1* misexpression (Fig. 3A, B), indicating that Ascl1 indeed actively represses the glutamatergic fate of ectopically produced neurons. In contrast, neither *Neurog2*, *NeuroD1* or *Atoh7* were able to interfere with the *Ascl1*-dependent induction of GABAergic neurons (Fig. 3A, C). Surprisingly, we found that embryos overexpressing both *Ascl1* and *Neurog2* displayed more ectopic *gad1*-positive cells compared to embryos injected with *Ascl1* alone (Fig. 3D), suggesting an unexpected synergistic effect. Altogether, these data demonstrate that, in this ectopic context, *Ascl1* is epitastic to these various bHLH genes. In the retina as well, overexpression of *Ascl1* together with *Neurog2* still resulted in an increased production of GABAergic cells within the ganglion cell layer (Fig. 4A, B). However, no synergy was observed in this context.

### *Ascl1* catecholaminergic inducing activity is impaired by glutamatergic factors

**Figure 5:**
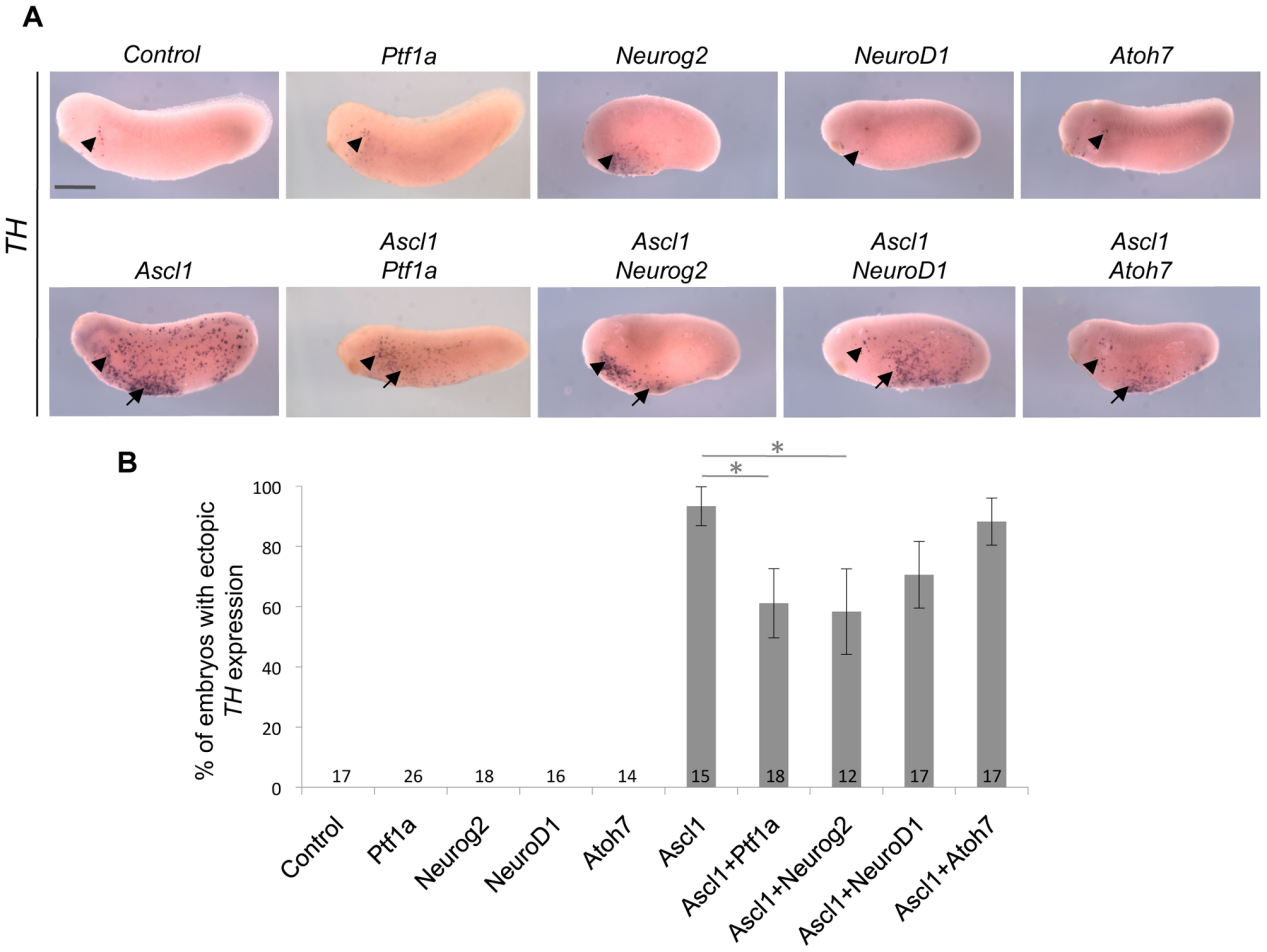
Comparison of the catecholaminergic inducing activities of five different bHLH factors. (A) Whole mount *in situ* hybridization analysis of *Tyrosine Hydroxylase (TH)* expression on stage 24 embryos injected with the indicated mRNAs in one blastomere at the two-cell stage (anterior on the left, dorsal side up). Arrowheads indicate the position of previously described *TH*-positive antero-ventral neurons [53], while arrows point to ectopic *TH* staining. Note that, among all tested bHLH factors, only *Ascl1* induces ectopic *TH* expression. Sibling embryos were also hybridized with the *tubb2b* probe as a positive control (see Figure 3A). (B) Quantification of embryos displaying ectopic *TH^+^* neurons, showing that *Neurog2* and *Ptf1a* significantly reduce the catecholaminergic inducing activity of *Ascl1*. Total number of analyzed embryos per condition is indicated in each bar. Error bars represent 95% confidence intervals. p<0,05 (*) (binomial test). Scale bar represents 500 μm.

As recently shown, *Ascl1* misexpression in the epidermis also leads to the ectopic generation of catecholaminergic neurons [53], authenticated by the expression of *tyrosine hydroxylase* (*TH*). We thus decided to pursue our comparative study by monitoring *TH* labelling after *Ascl1*, *Neurog2*, *NeuroD1*, *Atoh7* or *Ptf1a* mRNA injection. In contrast to *Ascl1*, none of these genes were able to promote the production of *TH*-positive neurons, emphasizing again the specific role of *Ascl1* in neuronal subtype determination (Fig. 5A). Epistatic analyses revealed that the proportion of embryos displaying ectopic *TH* expression was slightly decreased upon co-injection of *Ascl1* with either *Atoh7* or *NeuroD1*, although this was not statistically significant. It was however significantly reduced when *Ascl1* was misexpressed together with *Neurog2* or *Ptf1a*, suggesting that these two genes could interfere with the *Ascl1* catecholaminergic inducing activity (Fig. 5A, B). Such a result contrasts with the interaction previously demonstrated for the induction of GABAergic neurons and reflects that the same set of transcription factors establishes variable genetic interactions within distinct differentiation pathways.

### The GABAergic and catecholaminergic inducing activities of Ascl1 are conferred by its basic domain

**Figure 6:**
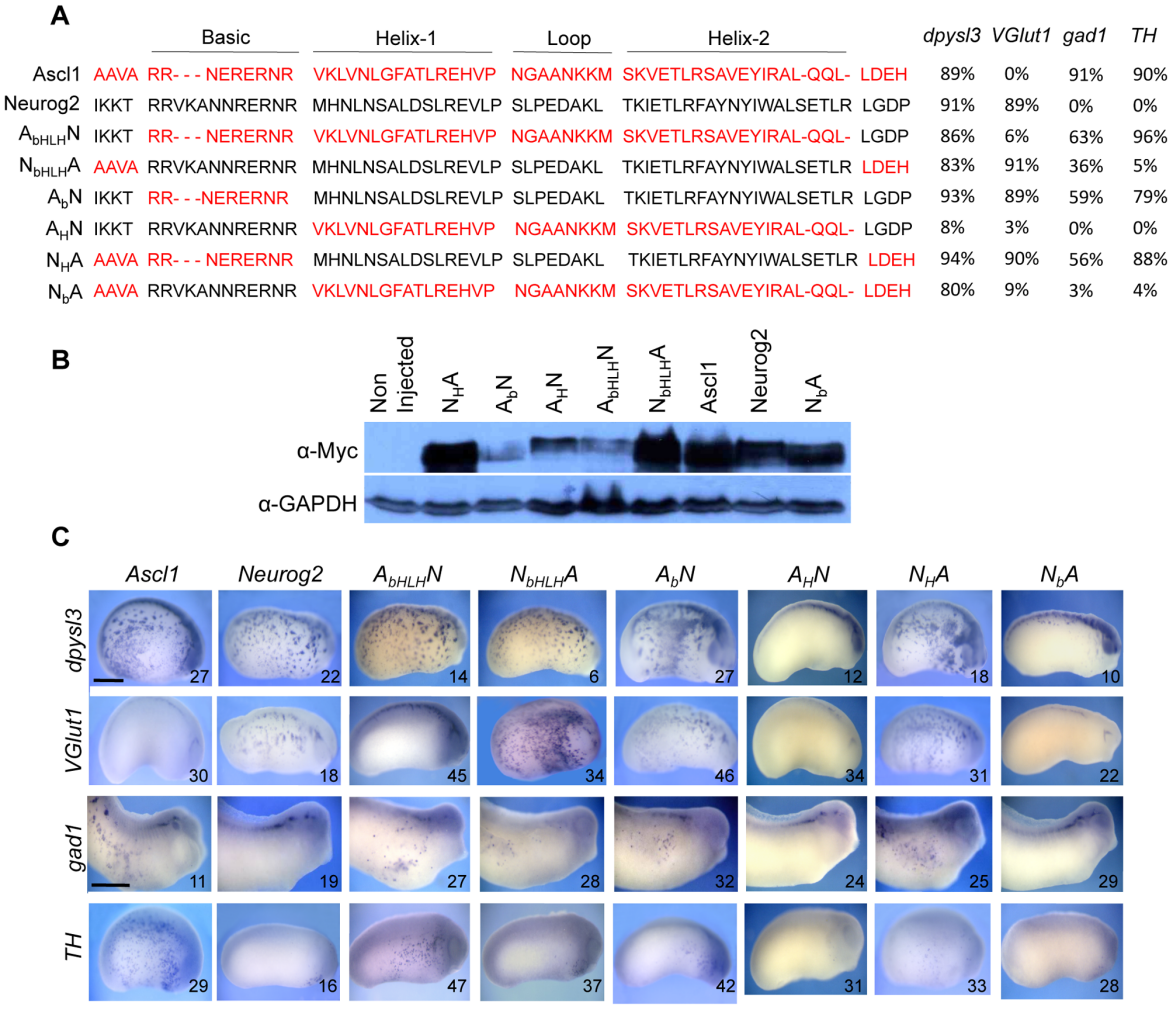
Mapping of Ascl1 and Neurog2 domains accounting for their functional specificity in neuronal subtype specification. (A) bHLH protein sequences of the Myc-tagged inducible Ascl1:Neurog2 chimeric constructs used in B and C. Ascl1 and Neurog2 sequences are depicted in red and black, respectively. Shown on the right and illustrated in (C) is the ability of the different proteins to induce *dpysl3*, *VGlut1, gad1* or *TH.* Percentages of embryos with ectopic expression are indicated. (B, C) Embryos were injected in one blastomere at the four-cell stage with the indicated wild-type or chimeric mRNA and dexamethasone was added at stage 13. (B) Western-blot using an anti-Myc antibody showing that each chimeric protein has been produced. Detection of GAPDH serves as a loading control. (C) Whole mount *in situ* hybridization analyses of *dpysl3*, *VGlut1, gad1* or *TH* expression on stage 22 or 28 injected embryos (anterior on the right, dorsal side up). Altogether, these domain-swapping experiments show that the GABAergic inducing activity of Ascl1 resides in its basic domain, while the glutamatergic inducing activity of Neurog2 is imparted by its HLH domain. In each panel the number of analysed embryos is indicated. Scale bar represents 250 μm.

We next examined which Ascl1 domains account for the protein activity in neuronal subtype specification. We used a series of chimeric *Ascl1:Neurog2* constructs (Fig. 6A) with interchanged basic and/or HLH domains (DNA binding and dimerization domain, respectively) and tested their ability to induce *TH*, *gad1* and *VGlut1* ectopic expression. Importantly, the chimeric proteins were properly expressed following mRNA injection (Fig. 6B) and all but one (A_H_N) retained a neurogenic activity, as inferred by their ability to promote the expression of the pan-neuronal marker *dpysl3* (previously called *CRMP4*; Fig. 6A, C). Noticeably, swapping the entire bHLH domains of Ascl1 and Neurog2 was sufficient to invert their respective properties in neurotransmitter phenotype induction. The Neurog2 protein containing the Ascl1 bHLH indeed converted into a GABAergic and catecholaminergic neuron inducer, while the Ascl1 protein with the Neurog2 bHLH turned into a glutamatergic one (Fig. 6A, C; compare A_bHLH_N to Ascl1 and N_bHLH_A to Neurog2). This demonstrates that the functional specificity of these two proteins resides in their respective bHLH domains. In addition, we found that the presence of the Ascl1 basic domain in chimeric constructs was necessary and sufficient to trigger *TH* and *gad1* ectopic expression (Fig. 6A, C; compare A_b_N and N_H_A to N_b_A for *gad1* and *TH* staining). Conversely, only the chimeric proteins containing the Neurog2 HLH domain had the ability to induce glutamatergic neurons (Fig. 6A, C; see *VGlut1* labelling for A_b_N and N_H_A *versus* N_b_A). In line with this observation, the two chimeric proteins containing both the basic domain of Ascl1 and the HLH of Neurog2 (A_b_N and N_H_A proteins) were able to simultaneously promote *gad1*, *TH* and *VGlut1* expression. Altogether, these results suggest that the functional specificities of Ascl1 and Neurog2 in neurotransmitter subtype specification do not reside in the same protein domain. GABAergic and catecholaminergic inducing activity of Ascl1 primarily relies on its basic domain while glutamatergic inducing activity of Neurog2 is imparted by its HLH domain.

**Figure 7:**
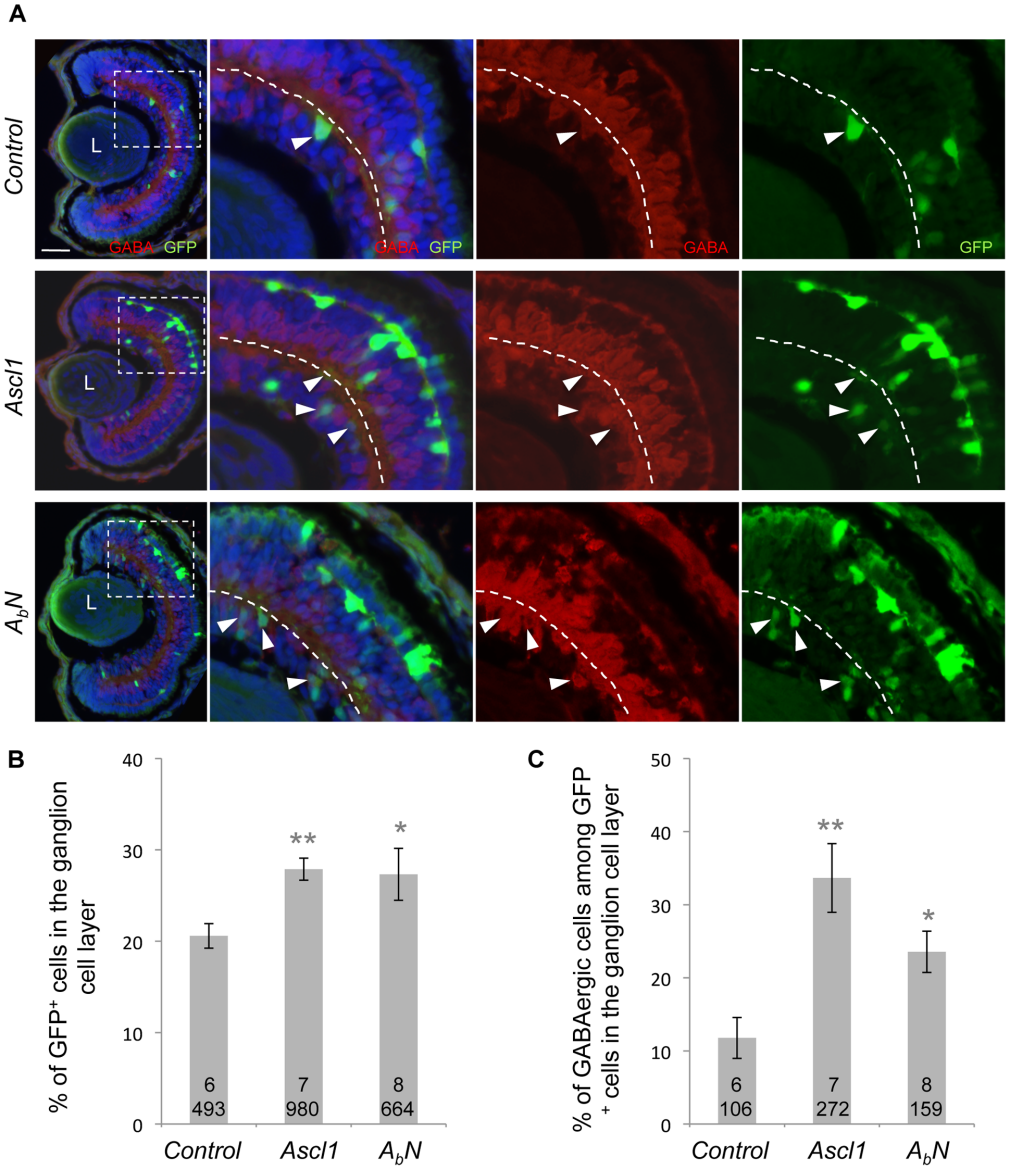
Retinal GABAergic inducing activity of Ascl1 resides in its basic domain. (A) Stage 41 retinal sections immunostained with anti-GABA (red) and anti-GFP (green) antibodies. Embryos were lipofected with *Ascl1* or the inducible *A_B_N* chimeric construct (encoding a Neurog2 protein with the basic domain of AsclI). Dexamethasone was added immediately after lipofection at stage 18. Panels on the right are higher magnifications of the dotted square delineated region. The dotted line indicates the inner plexiform layer. Arrowheads indicate GABA^+^/GFP^+^ cells within the ganglion cell layer. (B-C) Quantification of GFP^+^ cells and GABA^+^/GFP^+^ in the ganglion cell layer showing that similarly to *Ascl1*, *A_B_N* increases the percentage of cells in the ganglion cell layer but also biases these cells towards a GABAergic destiny. Total number of analyzed retinas and counted cells per condition is indicated in each bar. Values are given as mean +/– s.e.m. p<0,01 (**), p<0,05 (*) (Student’s t-test). L: Lens. Scale bar represents 50 μm.

To assess whether this also holds true within the retina, we overexpressed the chimeric construct *A_b_N* by *in vivo* lipofection (Fig. 7A). Similarly to wild-type *Ascl1*, *A_b_N* transfection increased the proportion of cells within the ganglion cell layer (Fig. 7B) and biased these additional neurons towards a GABAergic destiny (Fig. 7C). This indicates that Ascl1 basic domain is sufficient to drive a subset of retinal progenitor cells towards a GABAergic fate in the retina.

### *Phox2a* and *Hand2* are essential downstream components of Ascl1 catecholaminergic pathway but are dispensable for its GABAergic inducing activity

**Figure 8:**
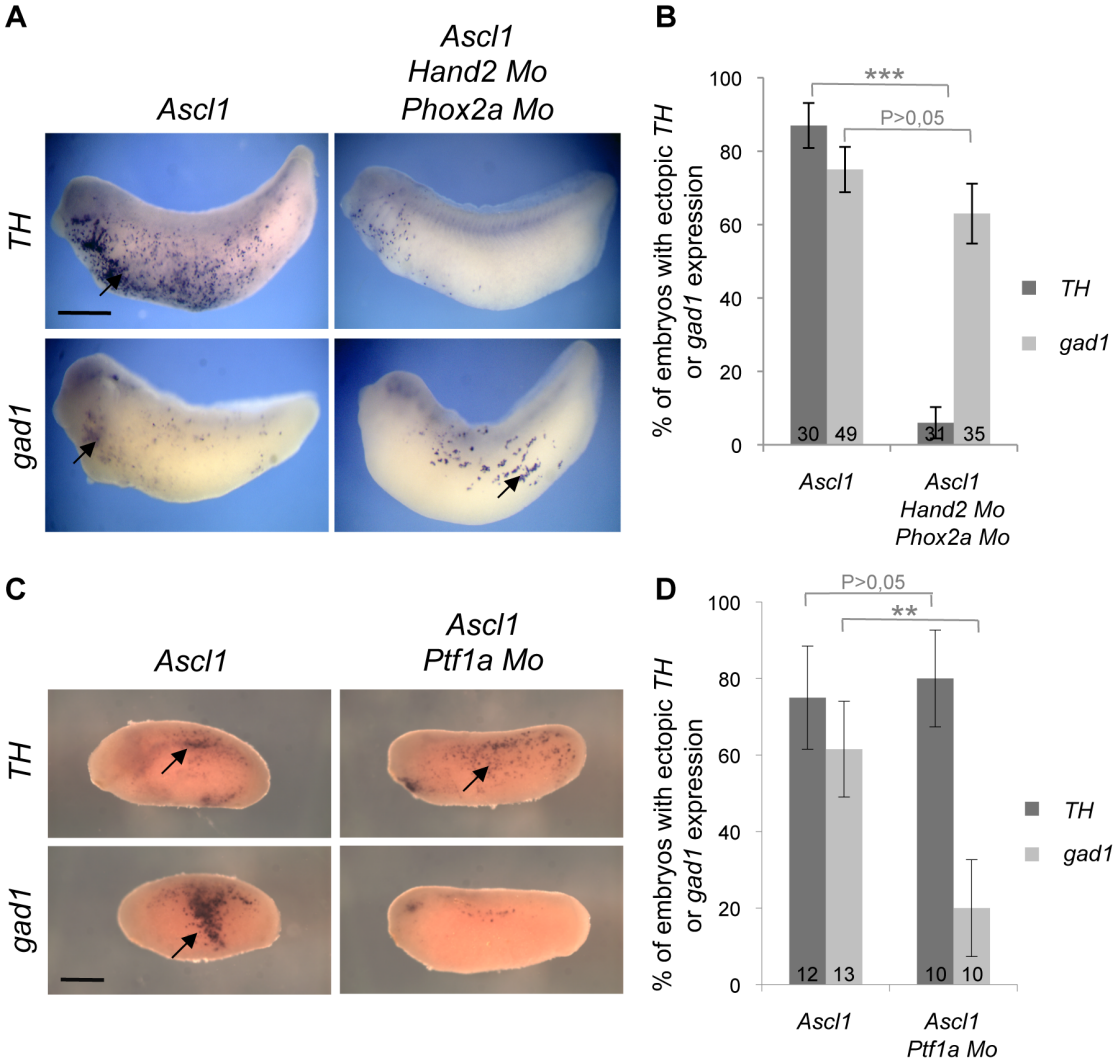
Distinct transcriptional networks sustain *Ascl1* GABAergic and catecholaminergic inducing activities. Whole mount *in situ* hybridization analyses of *TH* or *gad1* expression on stage 24 embryos following injection of mRNA and/or morpholinos (Mo), as indicated, in one blastomere at the two-cell stage. (A, C) Arrows point to ectopic *TH* or *gad1* expression (anterior on the left, dorsal side up). Sibling embryos were also hybridized with the *tubb2b* probe as a positive control (data not shown). (B, D) Quantification of embryos displaying ectopic *TH* or *gad1* expression. Note that *Phox2a* and *Hand2* knockdown significantly reduces Ascl1 ability to induce *TH* expression but does not impair its GABAergic inducing activity (B), which specifically depends on *Ptf1a* (D). Total number of analyzed embryos per condition is indicated in each bar. Error bars represent 95% confidence intervals. P<0,001 (***), p<0,01 (**) (binomial test). Scale bar represents 500 μm.

The DNA binding basic domain of Ascl1 being central to its activity, we next sought to identify potential transcriptional targets involved in its GABAergic *versus* catecholaminergic inducing activity. The homeodomain Phox2a and bHLH Hand2 proteins act downstream Ascl1 for the determination and differentiation of noradrenergic neurons [17], [19], [68], [69]. To examine whether these genes may also be involved in Ascl1 GABAergic inducing activity, we simultaneously misexpressed *Ascl1* while knocking down both *Phox2a* and *Hand2* using specific morpholinos. As previously described, this resulted in a significantly decreased proportion of embryos exhibiting ectopic *TH* staining. In contrast, the ability of Ascl1 to induce *gad1*-positive neurons appeared largely unaffected (Fig. 8A, B). This demonstrates that *Phox2a* and *Hand2*, although required for Ascl1-dependent production of catecholaminergic neurons, are dispensable for its GABAergic inducing activity.

### *Ptf1a* is required for Ascl1 GABAergic inducing activity as a direct transcriptional target

Since both *Ascl1* and *Ptf1a* share the ability to induce ectopic GABAergic neurons, in contrast to the other tested bHLH genes (Fig. 3), we next investigated whether they could act in the same transcriptional network. We first examined *Ptf1a* requirement for Ascl1 GABAergic and catecholaminergic inducing activities by simultaneously overexpressing *Ascl1* and knocking down *Ptf1a* (Fig. 8C, D). The *Ascl1*-dependent ectopic *TH* expression was unaffected by *Ptf1a* inhibition, showing that this gene is dispensable for Ascl1 catecholaminergic inducing activity. In contrast, the percentage of embryos displaying ectopic *gad1* staining was significantly decreased by the concomitant blockade of *Ptf1a*. These results indicate that *Ptf1a* acts downstream *Ascl1* for the generation of ectopic GABAergic neurons.

**Figure 9:**
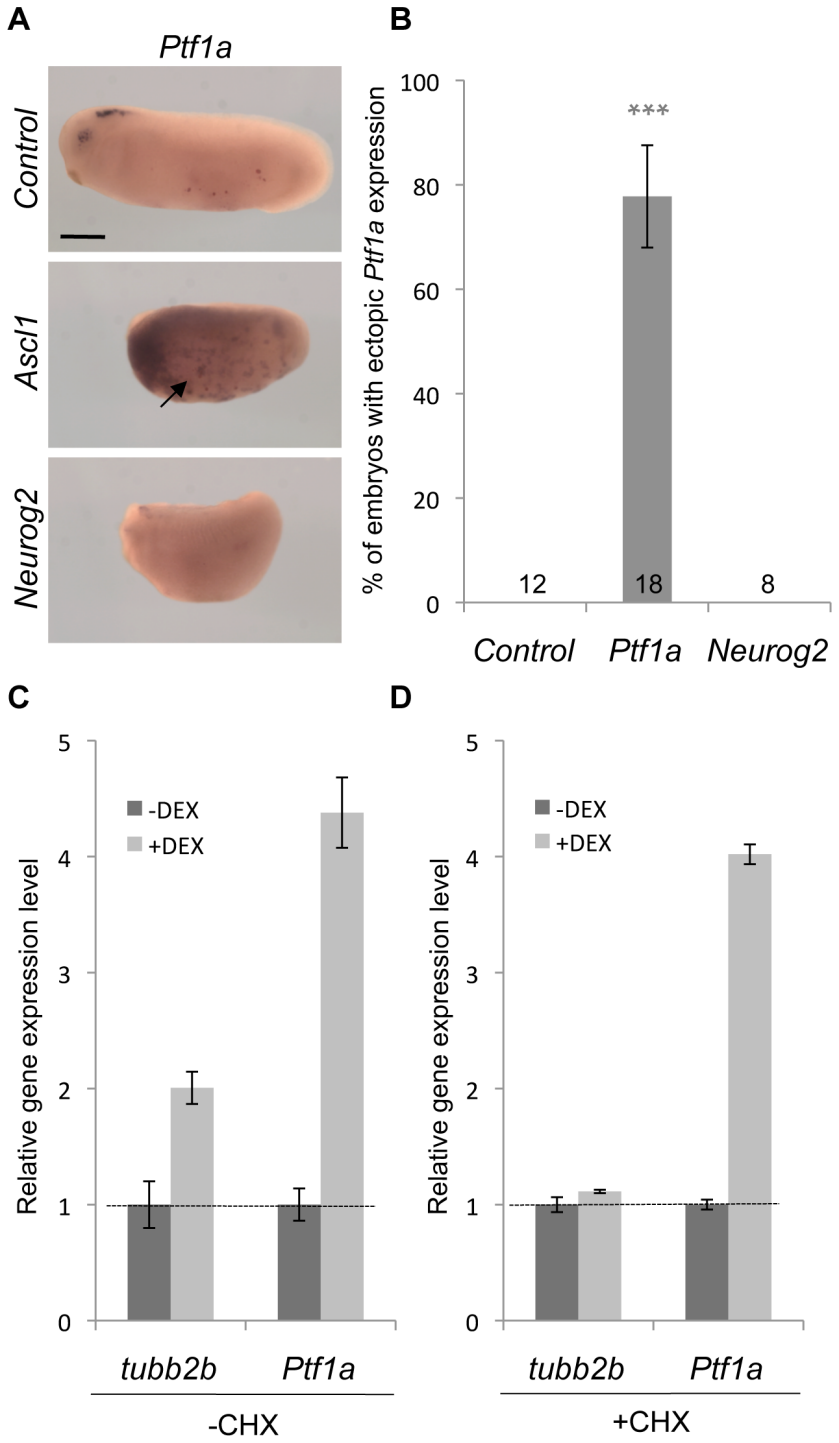
*Ptf1a* is a direct transcriptional target of *Ascl1*. (A) Whole mount *in situ* hybridization analyses of *Ptf1a* expression on stage 22 embryos injected with *Ascl1* or *Neurog2* mRNA in one blastomere at the two-cell stage. Note that Ascl1 is able to activate *Ptf1a* expression in the epidermis (arrow). Sibling embryos were also hybridized with the *tubb2b* probe as a positive control (data not shown). (B) Quantification of embryos displaying *Ptf1a* ectopic expression. Total number of analysed embryos per condition is indicated in each bar. Error bars represent 95% confidence intervals. P<0,001 (***) (binomial test). (C, D) RT-qPCR analyses of *tubb2b* and *Ptf1a* expression in embryos injected with *Ascl1-GR* at the two-cell stage and treated with dexamethasone (dex) and/or cycloheximide (CHX) from stage 19 to stage 21. Note that *Ptf1a* up-regulation upon dex treatment is maintained in the presence of CHX. Scale bar represents 500 μm.

We then tested whether this *Ptf1a* dependency might rely on a transcriptional interaction. We found that *Ascl1* mRNA injection promoted *Ptf1a* ectopic expression in the epidermis (Fig. 9A, B). In contrast, *Neurog2* misexpression was unable to do so. Together, these data suggest the existence of a positive transcriptional regulation exerted by Ascl1 on *Ptf1a*. We thus investigated whether *Ptf1a* could constitute a direct target of Ascl1. In this purpose, we performed gene induction assays on *Ascl1-GR* injected embryos in the absence of protein synthesis, using the translation-blocking drug cycloheximide (CHX). In the absence of CHX, *Ascl1* overexpression lead to a strong increase of both *tubb2b* and *Ptf1a* expression level, as assayed by RT-qPCR analysis (Fig. 9C). Only *Ptf1a* up-regulation persisted upon CHX treatment, consistent with *Ptf1a* being a direct Ascl1 target gene (Fig. 9D).

**Figure 10:**
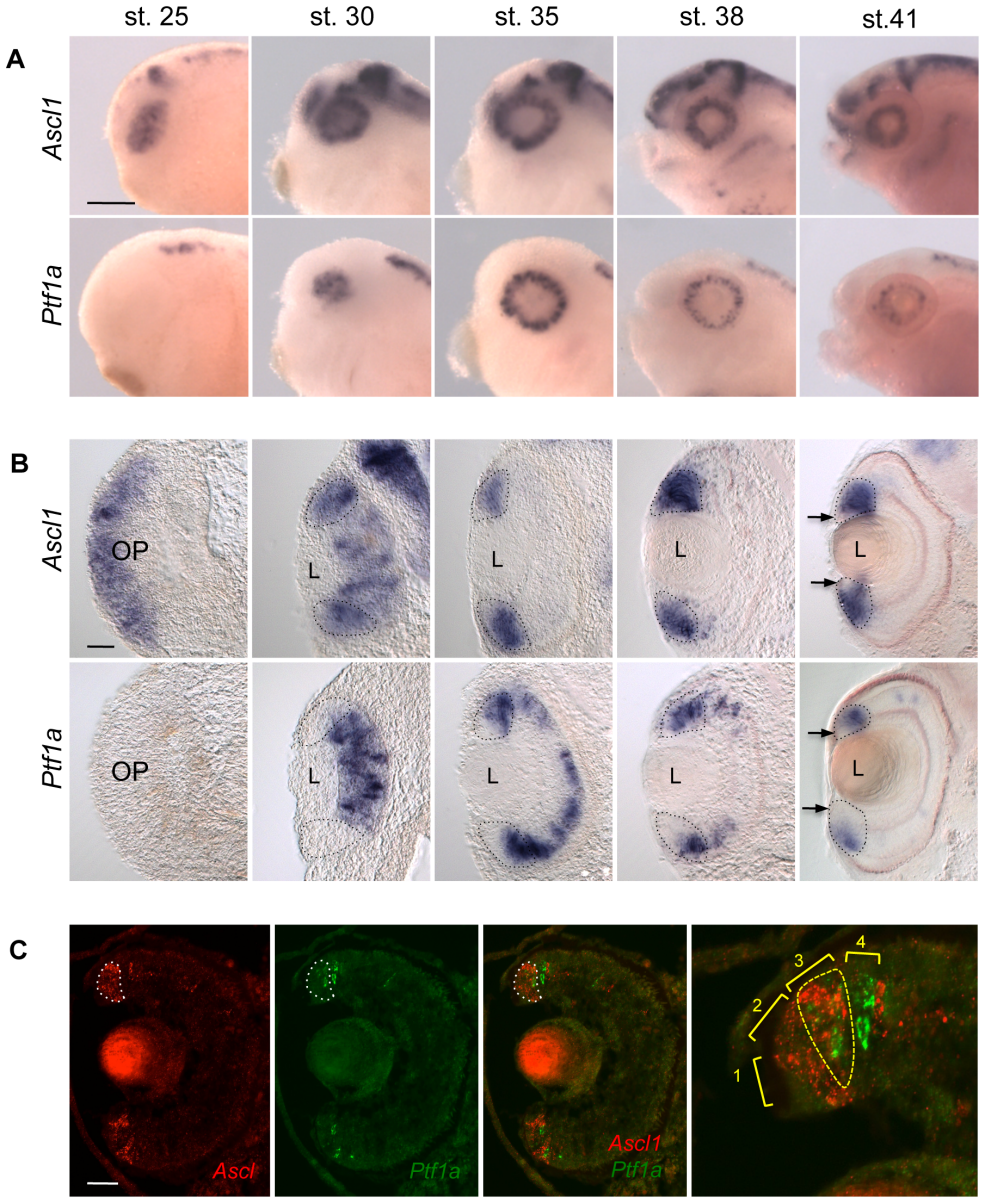
Comparison of *Ascl1* and *Ptf1a* expression patterns during retinogenesis. (A, B) Whole mount *in situ* hybridization analysis of *Ascl1* and *Ptf1a* expression during retinogenesis. Shown are representative pictures of the observed labelling in the head region (A; lateral views, anterior on the left) and on retinal cross sections (B; dorsal side up). *Ascl1* and *Ptf1a* are expressed in overlapping domains of the neural retina at stage 30 and in the ciliary marginal zone (CMZ, delineated by doted line) from stage 35 onwards. (C) Double fluorescent *in situ* hybridization against *Ptf1a* (green) and *Ascl1* (red) performed on stage 40 retinal sections. *Ascl1* staining in the CMZ is delineated by a white doted line. Panel on the right shows a magnification of the dorsal CMZ. Neither *Ascl1* nor *Ptf1a* are detected in zone 1 (stem cell compartment). In zone 2 (early progenitors), only *Ascl1* is expressed. The expression patterns of both genes overlap in zone 3 (late progenitors; yellow doted line). In zone 4 (postmitotic retinoblasts), *Ascl1* expression vanishes while that of *Ptf1a* persists. OP: Optic vesicle, L: Lens. Scale bar represents 300 μm (A) or 50 μm (B, C).

**Figure 11:**
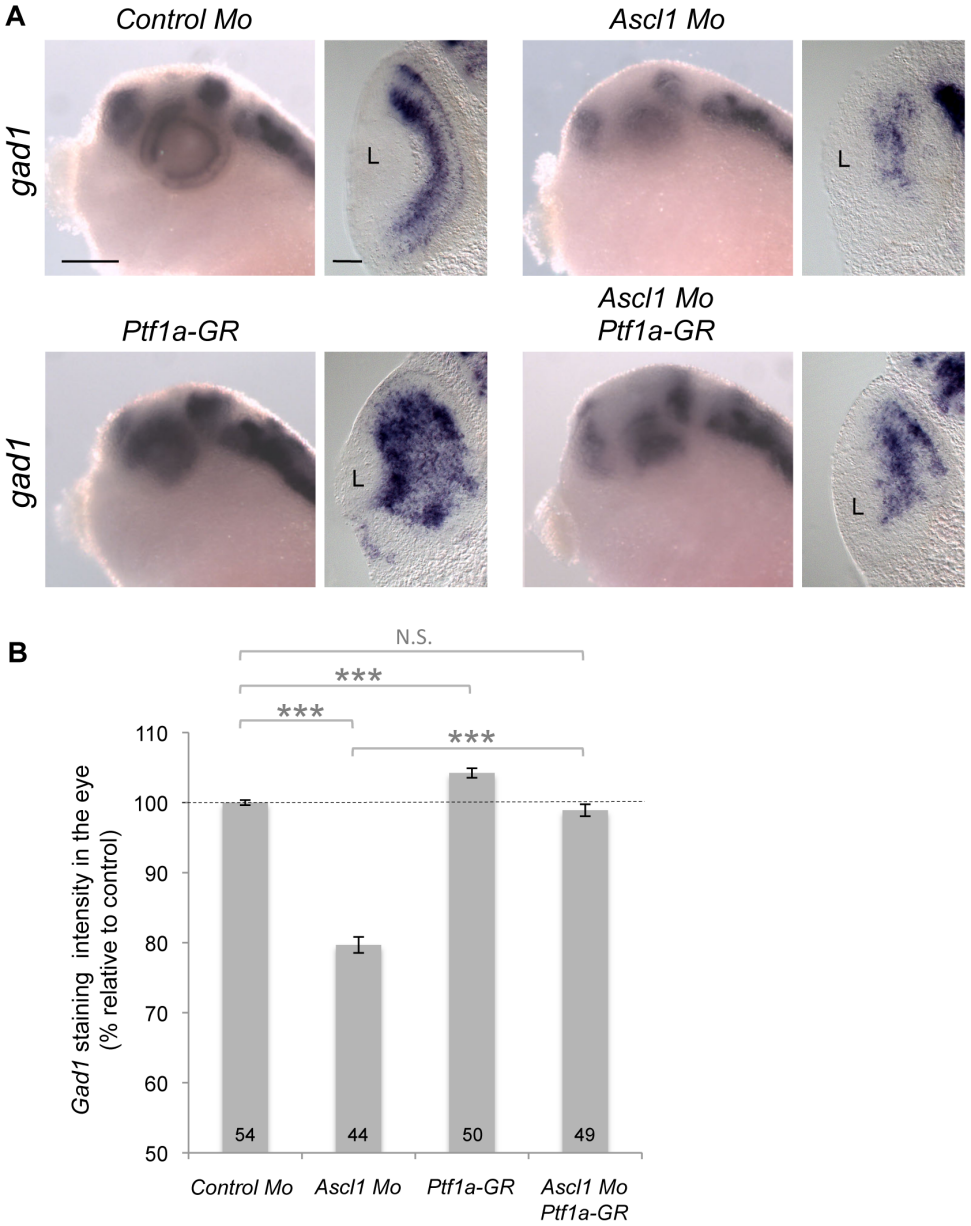
*Ptf1a* overexpression rescues *Ascl1* knockdown in the retina. (A) Whole mount *in situ* hybridization analysis of *gad 1* expression at stage 37. Embryos were injected with the indicated mRNAs and/or morpholinos (Mo) in two blastomeres at the two-cell stage and treated with dexamethasone at stage 22. Shown are representative pictures of the observed labelling in the head region (lateral views, anterior on the left) and on retinal sections (dorsal side up). (B) Quantification of *gad1* staining intensity in the eye of injected embryos. Results are presented as percentage increase/decrease relative to the average intensity found in controls. Total number of analyzed embryos per condition is indicated in each bar. Values are given as mean +/– s.e.m. p<0,001 (***) (Student’s t-test); N.S. : Non significant; L: Lens. Scale bar represents 300 μm (heads) or 50 μm (sections).

In order to know whether such transcriptional interaction could also hold true in the retina, we first carefully compared their expression pattern during retinogenesis (Fig. 10). Consistent with a *Ptf1a*-independent proneural function, we found that *Ascl1* was broadly expressed in early retinal precursors prior to the onset of *Ptf1a* expression. However, from stage 30 onwards, regions of overlapping expression were clearly observed in the neural retina of the optic cup and then within the neurogenic zone of the mature retina called the ciliary marginal zone (CMZ) [70]. We next asked whether *Ptf1a* overexpression could rescue *Ascl1* knockdown. We found indeed that *gad1* expression was restored to a control level in the retina of *Ascl1* morphant embryos overexpressing *Ptf1a* (Fig. 11). Altogether, these results are consistent with *Ascl*1 acting upstream *Ptf1a* in the transcriptional network controlling GABAergic neuron genesis in the retina.

## Discussion

**Figure 12:**
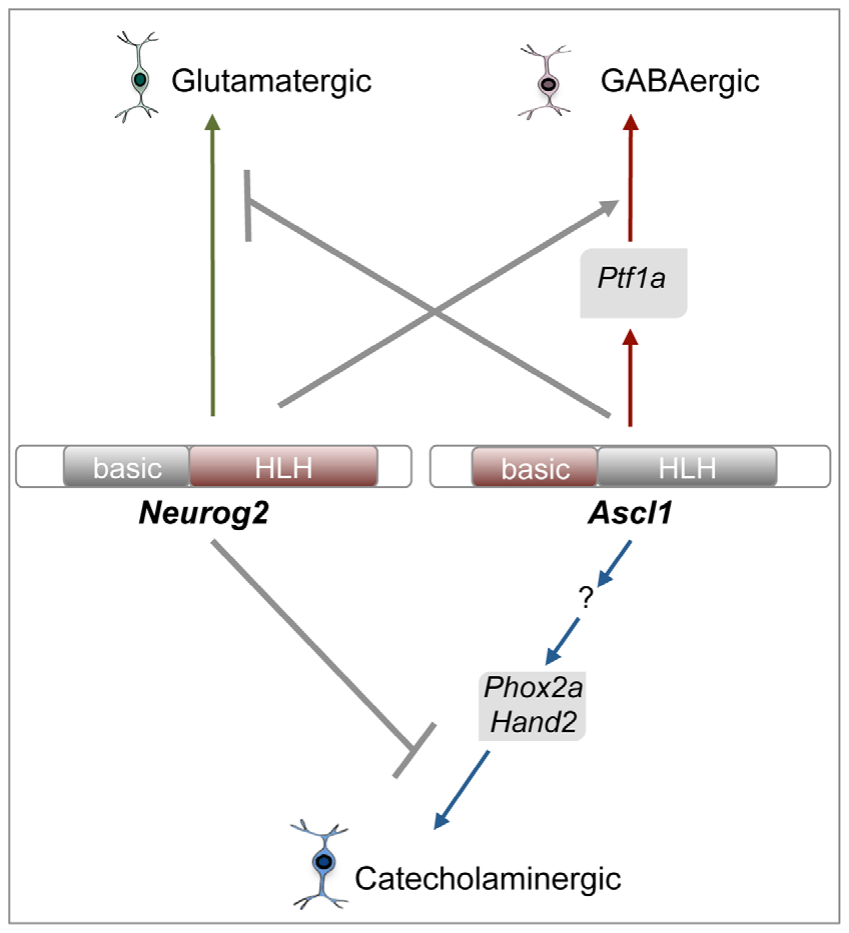
Summary of *Ascl1* genetic network in neuronal subtype specification. Green, red and blue arrows point to neuronal subtypes induced by *Neurog2* and *Ascl1*. Identified downstream components required for their respective activities are indicated. Grey arrows illustrate epistatic relationships between *Neurog2* and *Ascl1*, as suggested by our results. Protein domains in red confer to Ascl1 and Neurog2 their specific GABAergic/catecholaminergic and glutamatergic activities, respectively (see text for more details).

The molecular bases underlying neurotransmitter subtype determination in the retina remain largely unexplored. As previously shown in the brain and peripheral nervous system [6], [15], [18], [68], our data suggest that during retinogenesis, Ascl1 does not simply act as a proneural factor committing progenitor cells to a generic neuronal fate, but also contributes to the specification of their neurotransmitter identity. We showed indeed that Ascl1 is required for retinal GABAergic cell type genesis and sufficient to redirect a subpopulation of progenitors towards this inhibitory neuronal destiny. Besides, taking advantage of an *in vivo* neurogenic assay in the *Xenopus* epidermis, we found that Ascl1 GABAergic determining activity is instructive, as inferred by its ability to counteract glutamatergic differentiation programs. Finally, we gained insights into downstream genetic networks underlying Ascl1 functions in neurotransmitter subtype specification by showing that its GABAergic activity involves the direct transcriptional regulation of *Ptf1a* (Fig. 12).

### Ascl1 GABAergic determining activity in the retina

Most *Ascl1* gain and loss of function analyses in the retina have so far uncovered phenotypes predominantly related to its proneural activity. *Ascl1* has indeed been shown to be required for neuronal *versus* glial fate decisions during late neurogenesis [21], [22], [23], [71] and to participate, with other proneural genes, to the spatiotemporal progression of the neurogenic wave in the retina [72]. In line with this, recent lineage experiments in mouse revealed that *Ascl1*-expressing progenitors contribute to all major cell types of the retina with the exception of ganglion cells [3]. Such a primary function in determining a generic neuronal program has thus hindered the identification of its potential specific activity in retinal cell subtype specification. In line with this, overexpression of *Ascl1* or other bHLH genes such as *Neurog2* or *Atoh7* results in apparently similar cell type distribution defects within the *Xenopus* retina [46], [47], [55], [63]. In the present manuscript, we revealed that Ascl1 is actually endowed with a GABAergic determining function that superimposes to its proneural activity. Of note, neither *Neurog2* nor *Atoh7* exhibit this property. Importantly, our results are consistent with a previous study in chick reporting that, unlike *Neurog2*, *NeuroD1* or *Atoh7*, *Ascl1* misexpression promotes an overproduction of amacrine cells, the major retinal GABAergic cell type [73]. Therefore, we propose that Ascl1 not only promotes neurogenesis but also biases, in combination with other bHLH factors (see below), a subset of precursor cells towards particular neurotransmitter subtypes, thereby contributing to neuronal diversity during retinogenesis.

### Epistatic relationships of bHLH genes in neuronal subtype specification

The strong context-dependency of Ascl1 function in neurotransmitter subtype specification relies on the presence of other regionally expressed transcription factors including bHLH ones [2]. In line with this, our data show that Ascl1 ability to induce ectopic catecholaminergic neurons is impaired in the presence of Neurog2, NeuroD1 or Atoh7. In contrast, its GABAergic inducing activity is not counteracted by these factors, both ectopically and in retinal progenitors. Thus Ascl1 likely exerts an instructive role in promoting GABAergic cell fate as previously proposed in the ventral telencephalon [4], [6].

Unexpectedly, *Ascl1*-dependent ectopic GABAergic neuron production was found to be enhanced by *Neurog2* co-expression. This suggests that, in the few locations where both factors are co-expressed in the same cells [3], [74], [75], [76], they might act synergistically or at least cooperate within neuronal subtype specification programs. This could be the case in the retina where, although the *Ascl1* and *Neurog2* lineages are largely distinct, such progenitors expressing both genes have recently been described [3]. Interestingly, *Neurog2* was identified as a direct transcriptional target of *Ptf1a* in the chick dorsal spinal cord and cerebellum [77], two regions where *Ptf1a*, *Ascl1* and *Neurog2* expressions partially overlap [78]. *Neurog2* might thus participate within these domains in the *Ascl1*/*Ptf1a*-dependent GABAergic program. Additionally, Ascl1 and Neurog2 were shown to heterodimerize and function in common transcriptional complexes [77], [79], [80]. Investigating *Ascl1* interactions with other expressed bHLH genes in the retina will surely help understand the combinatorial codes governing neurotransmitter subtype determination.

### Functional domain and transcriptional network of Ascl1 involved in GABAergic cell determination

Previous reports proposed that the HLH domain of murine Ascl1 contains the information underlying its specific activity in neuronal subtype specification [20], [81]. In particular, a study using chimeric and mutated constructs concluded that Ascl1-mediated acquisition of GABAergic identity in forebrain precursor cultures does not occur through DNA binding, but rather through HLH domain protein-protein interactions [10]. The authors however raised the possibility that, in their assays, alterations of endogenous *Ascl1* expression may have interfered with proper GABAergic differentiation. Our domain swapping experiments led to an opposite conclusion, highlighting the essential role of Ascl1 basic domain in both its catecholaminergic and GABAergic inducing activities. Interestingly, the same was not true for Neurog2, whose ability to induce glutamatergic neurons was found to be imparted by its HLH domain. Although we cannot exclude that Ascl1 or Neurog2 might act through different molecular mechanisms depending on the cellular context, our data suggest that distinct domains within these bHLH factors mediate their functional specificities in neurotransmitter phenotype specification.

We showed that Ascl1 exerts its GABAergic and catecholaminergic determining activities by controlling distinct genetic cascades involving *Ptf1a* and *Phox2a*/*Hand2*, respectively. We found in particular that Ascl1 ability to promote *gad1*-positive neurons requires *Ptf1a*. Additionally, Ascl1 was able to activate *Ptf1a* transcription and to be necessary for its retinal expression. In line with such a positive transcriptional regulation, previous data showed that *Ascl1* is required for the induction and/or maintenance of *Ptf1a* during the late phase of dorsal sensory interneuron development [14], [82]. Moreover, we provide some evidence that *Ptf1a* might constitute a direct transcriptional target of Ascl1. Consensus binding sites for Ascl1 have previously been described in two conserved *Ptf1a* enhancer regions but have not proved to be functional [82]. Additional Ascl1 binding sites may however lie in other *Ptf1a* regulatory regions.

Altogether, our work demonstrated for the first time a role for Ascl1 in the generation of GABAergic interneurons in the retina and provided insights into the regulatory circuits responsible for its activity in neurotransmitter subtype specification.

## References

[1] Bertrand N, Castro DS, Guillemot F (2002) Proneural genes and the specification of neural cell types. Nat Rev Neurosci 3: 517–530. 12094208 10.1038/nrn874

[2] Powell LM, Jarman AP (2008) Context dependence of proneural bHLH proteins. Curr Opin Genet Dev 18: 411–417. 18722526 10.1016/j.gde.2008.07.012 PMC3287282

[3] Brzezinski JAt, Kim EJ, Johnson JE, Reh TA (2011) Ascl1 expression defines a subpopulation of lineage-restricted progenitors in the mammalian retina. Development 138: 3519–3531. 21771810 10.1242/dev.064006 PMC3143566

[4] Fode C, Ma Q, Casarosa S, Ang SL, Anderson DJ, et al (2000) A role for neural determination genes in specifying the dorsoventral identity of telencephalic neurons. Genes Dev 14: 67–80. 10640277 PMC316337

[5] Osorio J, Mueller T, Retaux S, Vernier P, Wullimann MF (2010) Phylotypic expression of the bHLH genes Neurogenin2, Neurod, and Mash1 in the mouse embryonic forebrain. J Comp Neurol 518: 851–871. 20058311 10.1002/cne.22247

[6] Parras CM, Schuurmans C, Scardigli R, Kim J, Anderson DJ, et al (2002) Divergent functions of the proneural genes Mash1 and Ngn2 in the specification of neuronal subtype identity. Genes Dev 16: 324–338. 11825874 10.1101/gad.940902 PMC155336

[7] Schuurmans C, Guillemot F (2002) Molecular mechanisms underlying cell fate specification in the developing telencephalon. Curr Opin Neurobiol 12: 26–34. 11861161 10.1016/s0959-4388(02)00286-6

[8] Wilkinson G, Dennis D, Schuurmans C (2013) Proneural genes in neocortical development. Neuroscience 253: 256–273. 23999125 10.1016/j.neuroscience.2013.08.029

[9] Casarosa S, Fode C, Guillemot F (1999) Mash1 regulates neurogenesis in the ventral telencephalon. Development 126: 525–534. 9876181 10.1242/dev.126.3.525

[10] Jo AY, Park CH, Aizawa S, Lee SH (2007) Contrasting and brain region-specific roles of neurogenin2 and mash1 in GABAergic neuron differentiation in vitro. Exp Cell Res 313: 4066–4081. 17936272 10.1016/j.yexcr.2007.08.026

[11] Schuurmans C, Armant O, Nieto M, Stenman JM, Britz O, et al (2004) Sequential phases of cortical specification involve Neurogenin-dependent and -independent pathways. EMBO J 23: 2892–2902. 15229646 10.1038/sj.emboj.7600278 PMC514942

[12] Wilson SW, Rubenstein JL (2000) Induction and dorsoventral patterning of the telencephalon. Neuron 28: 641–651. 11163256 10.1016/s0896-6273(00)00171-9

[13] Peltopuro P, Kala K, Partanen J (2010) Distinct requirements for Ascl1 in subpopulations of midbrain GABAergic neurons. Dev Biol 343: 63–70. 20417196 10.1016/j.ydbio.2010.04.015

[14] Mizuguchi R, Kriks S, Cordes R, Gossler A, Ma Q, et al (2006) Ascl1 and Gsh1/2 control inhibitory and excitatory cell fate in spinal sensory interneurons. Nat Neurosci 9: 770–778. 16715081 10.1038/nn1706

[15] Pattyn A, Simplicio N, van Doorninck JH, Goridis C, Guillemot F, et al (2004) Ascl1/Mash1 is required for the development of central serotonergic neurons. Nat Neurosci 7: 589–595. 15133515 10.1038/nn1247

[16] Jacob J, Ribes V, Moore S, Constable SC, Sasai N, et al.. (2013) Valproic Acid silencing of ascl1b/ascl1 results in the failure of serotonergic differentiation in a zebrafish model of Fetal Valproate Syndrome. Dis Model Mech. 10.1242/dmm.013219 PMC3882053 24135485

[17] Goridis C, Rohrer H (2002) Specification of catecholaminergic and serotonergic neurons. Nat Rev Neurosci 3: 531–541. 12094209 10.1038/nrn871

[18] Hirsch MR, Tiveron MC, Guillemot F, Brunet JF, Goridis C (1998) Control of noradrenergic differentiation and Phox2a expression by MASH1 in the central and peripheral nervous system. Development 125: 599–608. 9435281 10.1242/dev.125.4.599

[19] Howard MJ (2005) Mechanisms and perspectives on differentiation of autonomic neurons. Dev Biol 277: 271–286. 15617674 10.1016/j.ydbio.2004.09.034

[20] Park CH, Kang JS, Kim JS, Chung S, Koh JY, et al (2006) Differential actions of the proneural genes encoding Mash1 and neurogenins in Nurr1-induced dopamine neuron differentiation. J Cell Sci 119: 2310–2320. 16723737 10.1242/jcs.02955

[21] Nelson BR, Hartman BH, Ray CA, Hayashi T, Bermingham-McDonogh O, et al (2009) Acheate-scute like 1 (Ascl1) is required for normal delta-like (Dll) gene expression and notch signaling during retinal development. Dev Dyn 238: 2163–2178. 19191219 10.1002/dvdy.21848 PMC2905851

[22] Tomita K, Moriyoshi K, Nakanishi S, Guillemot F, Kageyama R (2000) Mammalian achaete-scute and atonal homologs regulate neuronal versus glial fate determination in the central nervous system. EMBO J 19: 5460–5472. 11032813 10.1093/emboj/19.20.5460 PMC314003

[23] Tomita K, Nakanishi S, Guillemot F, Kageyama R (1996) Mash1 promotes neuronal differentiation in the retina. Genes Cells 1: 765–774. 9077445 10.1111/j.1365-2443.1996.tb00016.x

[24] Jasoni CL, Reh TA (1996) Temporal and spatial pattern of MASH-1 expression in the developing rat retina demonstrates progenitor cell heterogeneity. J Comp Neurol 369: 319–327. 8727003 10.1002/(SICI)1096-9861(19960527)369:2<319::AID-CNE11>3.0.CO;2-C

[25] Jasoni CL, Walker MB, Morris MD, Reh TA (1994) A chicken achaete-scute homolog (CASH-1) is expressed in a temporally and spatially discrete manner in the developing nervous system. Development 120: 769–783. 7600956 10.1242/dev.120.4.769

[26] Marquardt T, Ashery-Padan R, Andrejewski N, Scardigli R, Guillemot F, et al (2001) Pax6 is required for the multipotent state of retinal progenitor cells. Cell 105: 43–55. 11301001 10.1016/s0092-8674(01)00295-1

[27] Agathocleous M, Harris WA (2009) From progenitors to differentiated cells in the vertebrate retina. Annu Rev Cell Dev Biol 25: 45–69. 19575661 10.1146/annurev.cellbio.042308.113259

[28] Hatakeyama J, Kageyama R (2004) Retinal cell fate determination and bHLH factors. Semin Cell Dev Biol 15: 83–89. 15036211 10.1016/j.semcdb.2003.09.005

[29] Ohsawa R, Kageyama R (2008) Regulation of retinal cell fate specification by multiple transcription factors. Brain Res 1192: 90–98. 17488643 10.1016/j.brainres.2007.04.014

[30] Adler R, Raymond PA (2008) Have we achieved a unified model of photoreceptor cell fate specification in vertebrates? Brain Res 1192: 134–150. 17466954 10.1016/j.brainres.2007.03.044 PMC2288638

[31] Feng L, Xie ZH, Ding Q, Xie X, Libby RT, et al (2010) MATH5 controls the acquisition of multiple retinal cell fates. Mol Brain 3: 36. 21087508 10.1186/1756-6606-3-36 PMC2994854

[32] Mo Z, Li S, Yang X, Xiang M (2004) Role of the Barhl2 homeobox gene in the specification of glycinergic amacrine cells. Development 131: 1607–1618. 14998930 10.1242/dev.01071

[33] Suga A, Taira M, Nakagawa S (2009) LIM family transcription factors regulate the subtype-specific morphogenesis of retinal horizontal cells at post-migratory stages. Dev Biol 330: 318–328. 19361492 10.1016/j.ydbio.2009.04.002

[34] Zaghloul NA, Moody SA (2007) Changes in Rx1 and Pax6 activity at eye field stages differentially alter the production of amacrine neurotransmitter subtypes in Xenopus. Mol Vis 13: 86–95. 17277735 PMC2503186

[35] Feng L, Xie X, Joshi PS, Yang Z, Shibasaki K, et al (2006) Requirement for Bhlhb5 in the specification of amacrine and cone bipolar subtypes in mouse retina. Development 133: 4815–4825. 17092954 10.1242/dev.02664 PMC2992969

[36] Duquette PM, Zhou X, Yap NL, MacLaren EJ, Lu JJ, et al (2010) Loss of LMO4 in the retina leads to reduction of GABAergic amacrine cells and functional deficits. PLoS One 5: e13232. 20949055 10.1371/journal.pone.0013232 PMC2951357

[37] Jiang H, Xiang M (2009) Subtype specification of GABAergic amacrine cells by the orphan nuclear receptor Nr4a2/Nurr1. J Neurosci 29: 10449–10459. 19692620 10.1523/JNEUROSCI.3048-09.2009 PMC2751734

[38] Lang B, Zhao L, Cai L, McKie L, Forrester JV, et al (2010) GABAergic amacrine cells and visual function are reduced in PAC1 transgenic mice. Neuropharmacology 58: 215–225. 19596361 10.1016/j.neuropharm.2009.07.003

[39] Dullin JP, Locker M, Robach M, Henningfeld KA, Parain K, et al (2007) Ptf1a triggers GABAergic neuronal cell fates in the retina. BMC Dev Biol 7: 110. 17910758 10.1186/1471-213X-7-110 PMC2212653

[40] Fujitani Y, Fujitani S, Luo H, Qiu F, Burlison J, et al (2006) Ptf1a determines horizontal and amacrine cell fates during mouse retinal development. Development 133: 4439–4450. 17075007 10.1242/dev.02598

[41] Jusuf PR, Almeida AD, Randlett O, Joubin K, Poggi L, et al (2011) Origin and determination of inhibitory cell lineages in the vertebrate retina. J Neurosci 31: 2549–2562. 21325522 10.1523/JNEUROSCI.4713-10.2011 PMC3083844

[42] Lelievre EC, Lek M, Boije H, Houille-Vernes L, Brajeul V, et al (2011) Ptf1a/Rbpj complex inhibits ganglion cell fate and drives the specification of all horizontal cell subtypes in the chick retina. Dev Biol 358: 296–308. 21839069 10.1016/j.ydbio.2011.07.033

[43] Nakhai H, Sel S, Favor J, Mendoza-Torres L, Paulsen F, et al (2007) Ptf1a is essential for the differentiation of GABAergic and glycinergic amacrine cells and horizontal cells in the mouse retina. Development 134: 1151–1160. 17301087 10.1242/dev.02781

[44] Ferreiro B, Skoglund P, Bailey A, Dorsky R, Harris WA (1993) XASH1, a Xenopus homolog of achaete-scute: a proneural gene in anterior regions of the vertebrate CNS. Mech Dev 40: 25–36. 8443105 10.1016/0925-4773(93)90085-c

[45] Afelik S, Chen Y, Pieler T (2006) Combined ectopic expression of Pdx1 and Ptf1a/p48 results in the stable conversion of posterior endoderm into endocrine and exocrine pancreatic tissue. Genes Dev 20: 1441–1446. 16751182 10.1101/gad.378706 PMC1475757

[46] Perron M, Opdecamp K, Butler K, Harris WA, Bellefroid EJ (1999) X-ngnr-1 and Xath3 promote ectopic expression of sensory neuron markers in the neurula ectoderm and have distinct inducing properties in the retina. Proc Natl Acad Sci U S A 96: 14996–15001. 10611326 10.1073/pnas.96.26.14996 PMC24761

[47] Kanekar S, Perron M, Dorsky R, Harris WA, Jan LY, et al (1997) Xath5 participates in a network of bHLH genes in the developing Xenopus retina. Neuron 19: 981–994. 9390513 10.1016/s0896-6273(00)80391-8

[48] Chitnis A, Kintner C (1996) Sensitivity of proneural genes to lateral inhibition affects the pattern of primary neurons in Xenopus embryos. Development 122: 2295–2301. 8681809 10.1242/dev.122.7.2295

[49] Chalfie M, Tu Y, Euskirchen G, Ward WW, Prasher DC (1994) Green fluorescent protein as a marker for gene expression. Science 263: 802–805. 8303295 10.1126/science.8303295

[50] Talikka M, Perez SE, Zimmerman K (2002) Distinct patterns of downstream target activation are specified by the helix-loop-helix domain of proneural basic helix-loop-helix transcription factors. Dev Biol 247: 137–148. 12074558 10.1006/dbio.2002.0677

[51] Gammill LS, Sive H (1997) Identification of otx2 target genes and restrictions in ectodermal competence during Xenopus cement gland formation. Development 124: 471–481. 9053323 10.1242/dev.124.2.471

[52] Nieuwkoop PD (1967) The "organization centre". 3. Segregation and pattern formation in morphogenetic fields. Acta Biotheor 17: 178–194. 4967351 10.1007/BF01601987

[53] Parlier D, Ariza A, Christulia F, Genco F, Vanhomwegen J, et al (2008) Xenopus zinc finger transcription factor IA1 (Insm1) expression marks anteroventral noradrenergic neuron progenitors in Xenopus embryos. Dev Dyn 237: 2147–2157. 18627098 10.1002/dvdy.21621

[54] Holt CE, Garlick N, Cornel E (1990) Lipofection of cDNAs in the embryonic vertebrate central nervous system. Neuron 4: 203–214. 1689586 10.1016/0896-6273(90)90095-w

[55] Ohnuma S, Mann F, Boy S, Perron M, Harris WA (2002) Lipofection strategy for the study of Xenopus retinal development. Methods 28: 411–419. 12507459 10.1016/s1046-2023(02)00260-8

[56] Li M, Sipe CW, Hoke K, August LL, Wright MA, et al (2006) The role of early lineage in GABAergic and glutamatergic cell fate determination in Xenopus laevis. J Comp Neurol 495: 645–657. 16506195 10.1002/cne.20900

[57] Amato MA, Boy S, Arnault E, Girard M, Della Puppa A, et al (2005) Comparison of the expression patterns of five neural RNA binding proteins in the Xenopus retina. J Comp Neurol 481: 331–339. 15593335 10.1002/cne.20387

[58] Souopgui J, Klisch TJ, Pieler T, Henningfeld KA (2007) Expression and regulation of Xenopus CRMP-4 in the developing nervous system. Int J Dev Biol 51: 339–343. 17554687 10.1387/ijdb.062235js

[59] Parain K, Mazurier N, Bronchain O, Borday C, Cabochette P, et al (2012) A large scale screen for neural stem cell markers in Xenopus retina. Dev Neurobiol 72: 491–506. 22275214 10.1002/dneu.20973

[60] Lea R, Bonev B, Dubaissi E, Vize PD, Papalopulu N (2012) Multicolor fluorescent in situ mRNA hybridization (FISH) on whole mounts and sections. Methods Mol Biol 917: 431–444. 22956102 10.1007/978-1-61779-992-1_24

[61] Taelman V, Van Wayenbergh R, Solter M, Pichon B, Pieler T, et al (2004) Sequences downstream of the bHLH domain of the Xenopus hairy-related transcription factor-1 act as an extended dimerization domain that contributes to the selection of the partners. Dev Biol 276: 47–63. 15531363 10.1016/j.ydbio.2004.08.019

[62] Farah MH (2006) Neurogenesis and cell death in the ganglion cell layer of vertebrate retina. Brain Res Rev 52: 264–274. 16764935 10.1016/j.brainresrev.2006.04.002

[63] Wang JC, Harris WA (2005) The role of combinational coding by homeodomain and bHLH transcription factors in retinal cell fate specification. Dev Biol 285: 101–115. 16040025 10.1016/j.ydbio.2005.05.041

[64] Lee JE, Hollenberg SM, Snider L, Turner DL, Lipnick N, et al (1995) Conversion of Xenopus ectoderm into neurons by NeuroD, a basic helix-loop-helix protein. Science 268: 836–844. 7754368 10.1126/science.7754368

[65] Ma Q, Kintner C, Anderson DJ (1996) Identification of neurogenin, a vertebrate neuronal determination gene. Cell 87: 43–52. 8858147 10.1016/s0092-8674(00)81321-5

[66] Rupp RA, Snider L, Weintraub H (1994) Xenopus embryos regulate the nuclear localization of XMyoD. Genes Dev 8: 1311–1323. 7926732 10.1101/gad.8.11.1311

[67] Turner DL, Weintraub H (1994) Expression of achaete-scute homolog 3 in Xenopus embryos converts ectodermal cells to a neural fate. Genes Dev 8: 1434–1447. 7926743 10.1101/gad.8.12.1434

[68] Lo L, Tiveron MC, Anderson DJ (1998) MASH1 activates expression of the paired homeodomain transcription factor Phox2a, and couples pan-neuronal and subtype-specific components of autonomic neuronal identity. Development 125: 609–620. 9435282 10.1242/dev.125.4.609

[69] Morikawa Y, D'Autreaux F, Gershon MD, Cserjesi P (2007) Hand2 determines the noradrenergic phenotype in the mouse sympathetic nervous system. Dev Biol 307: 114–126. 17531968 10.1016/j.ydbio.2007.04.027 PMC1952239

[70] Perron M, Kanekar S, Vetter ML, Harris WA (1998) The genetic sequence of retinal development in the ciliary margin of the Xenopus eye. Dev Biol 199: 185–200. 9698439 10.1006/dbio.1998.8939

[71] Hatakeyama J, Tomita K, Inoue T, Kageyama R (2001) Roles of homeobox and bHLH genes in specification of a retinal cell type. Development 128: 1313–1322. 11262232 10.1242/dev.128.8.1313

[72] Hufnagel RB, Le TT, Riesenberg AL, Brown NL (2010) Neurog2 controls the leading edge of neurogenesis in the mammalian retina. Dev Biol 340: 490–503. 20144606 10.1016/j.ydbio.2010.02.002 PMC2854206

[73] Mao W, Yan RT, Wang SZ (2009) Proneural gene ash1 promotes amacrine cell production in the chick retina. Dev Neurobiol 69: 88–104. 19067322 10.1002/dneu.20693 PMC2629813

[74] Kele J, Simplicio N, Ferri AL, Mira H, Guillemot F, et al (2006) Neurogenin 2 is required for the development of ventral midbrain dopaminergic neurons. Development 133: 495–505. 16410412 10.1242/dev.02223

[75] Mizuguchi R, Sugimori M, Takebayashi H, Kosako H, Nagao M, et al (2001) Combinatorial roles of olig2 and neurogenin2 in the coordinated induction of pan-neuronal and subtype-specific properties of motoneurons. Neuron 31: 757–771. 11567615 10.1016/s0896-6273(01)00413-5

[76] Roybon L, Deierborg T, Brundin P, Li JY (2009) Involvement of Ngn2, Tbr and NeuroD proteins during postnatal olfactory bulb neurogenesis. Eur J Neurosci 29: 232–243. 19200230 10.1111/j.1460-9568.2008.06595.x

[77] Henke RM, Savage TK, Meredith DM, Glasgow SM, Hori K, et al (2009) Neurog2 is a direct downstream target of the Ptf1a-Rbpj transcription complex in dorsal spinal cord. Development 136: 2945–2954. 19641016 10.1242/dev.035352 PMC2723066

[78] Zordan P, Croci L, Hawkes R, Consalez GG (2008) Comparative analysis of proneural gene expression in the embryonic cerebellum. Dev Dyn 237: 1726–1735. 18498101 10.1002/dvdy.21571

[79] Gradwohl G, Fode C, Guillemot F (1996) Restricted expression of a novel murine atonal-related bHLH protein in undifferentiated neural precursors. Dev Biol 180: 227–241. 8948587 10.1006/dbio.1996.0297

[80] Henke RM, Meredith DM, Borromeo MD, Savage TK, Johnson JE (2009) Ascl1 and Neurog2 form novel complexes and regulate Delta-like3 (Dll3) expression in the neural tube. Dev Biol 328: 529–540. 19389376 10.1016/j.ydbio.2009.01.007 PMC2698949

[81] Nakada Y, Hunsaker TL, Henke RM, Johnson JE (2004) Distinct domains within Mash1 and Math1 are required for function in neuronal differentiation versus neuronal cell-type specification. Development 131: 1319–1330. 14993186 10.1242/dev.01008

[82] Meredith DM, Masui T, Swift GH, MacDonald RJ, Johnson JE (2009) Multiple transcriptional mechanisms control Ptf1a levels during neural development including autoregulation by the PTF1-J complex. J Neurosci 29: 11139–11148. 19741120 10.1523/JNEUROSCI.2303-09.2009 PMC2758856

